# High prevalence of gastrointestinal parasites in dogs from Saipan, Northern Mariana Islands, including the zoonotic *Ancylostoma ceylanicum*

**DOI:** 10.1186/s13071-026-07258-8

**Published:** 2026-01-28

**Authors:** Maureen A. Kelly, Kris Anderson, Pablo D. Jimenez Castro, Christian Savard, Samantha Loo, Jeffrey Tereski, Christian M. Leutenegger, Guilherme G. Verocai

**Affiliations:** 1 Department of Veterinary Pathobiology, College of Veterinary Medicine and Biological Sciences, Texas A&M University, College Station, TX 77843 USA; 2 Equine Mobile Veterinary Services, Santa Fe, TX 77510 USA; 3 Antech Diagnostics, Loveland, CO 80538 USA; 4 Biovet Inc. (an Antech Diagnostics of Mars Petcare Science & Diagnostics Company), Saint-Hyacinthe, Québec, J2S 8WS Canada; 5 Grupo de Parasitologia Veterinaria, Universidad Nacional de Colombia, Bogota, Colombia

**Keywords:** *Ancylostoma*, Cutaneous larval migrans, *Giardia duodenalis*, Hookworm, Parasitic zoonoses, Soil-transmitted helminths, *Toxocara*, Visceral larva migrans

## Abstract

**Background:**

Gastrointestinal (GI) parasites of dogs, including helminths and protozoans, are of substantial relevance to veterinary medicine and public health. Nevertheless, epidemiological data are scarce worldwide, especially in remote locations. The emergence of novel technologies and diagnostic platforms facilitates comprehensive screening of multiple GI parasites. Our study aims to establish a baseline prevalence for GI parasites in dogs from Saipan, Northern Mariana Islands.

**Methods:**

Fecal samples were collected from dogs (*n* = 420) from May to June 2023 during a spay-neuter campaign. Age, sex, ownership status, and residing location were recorded. Following genomic extraction, samples were screened using the KeyScreen™ GI Parasite PCR (Antech Diagnostics), a real-time PCR panel that detects 20 endoparasite infections, detects benzimidazole resistance in *Ancylostoma caninum*, and determines the zoonotic potential of *Giardia duodenalis*. If inconclusive results for *Ancylostoma* spp. were obtained, conventional PCR and Sanger sequencing were performed, targeting the ITS-1 region for species identification. Additionally, demographics (i.e. age, sex, ownership, and residing location) were evaluated as potential risk factors for each pathogen as the outcome with an initial univariate analysis, followed by multivariable logistic regression with backward stepwise selection.

**Results:**

Overall, parasites were detected in 267/420 (63.5%; 95% CI: 58.7–68.1) canine samples. The most detected parasite genus was *Ancylostoma* spp. (*n* = 224; 53.3%), followed by *G. duodenalis* (*n* = 67; 15.9%), *Trichuris* (*n* = 39; 9.2%), *Dipylidium* (*n* = 25; 5.9%), *Toxocara* (*n* = 15; 3.5%), *Cystoisospora* (*n* = 10; 2.3%), and *Cryptosporidium* (*n* = 5; 1.1%). Assemblages with zoonotic potential of *G. duodenalis* and the SNPs 167Y and 134H in the isotype 1 Beta-tubulin gene associated with benzimidazole-resistance in *A. caninum* were not detected. Risk factors significantly associated with infection were age, district, and ownership with *Trichuris*; age and ownership with *Ancylostoma*, *Giardia*, and *Dipylidium*; and ownership with *Toxocara* and *Cystoisospora*. Hookworm-positive samples were further characterized to species level. Overall, *Ancylostoma caninum* and *A. ceylanicum*/*A. duodenale* were confirmed in 196 (46.7%) and 57 (13.5%) dogs, respectively. Further sequencing confirmed the presence of zoonotic *A. ceylanicum* in at least 21 samples, approximately 5% of the sampled dog population, distributed geographically across all districts.

**Conclusions:**

To our knowledge, our study is the first to provide epidemiological data on canine gastrointestinal parasites in Saipan. The high prevalence of multiple parasites of One Health importance reinforces the need for surveillance and the implementation of prevention and control strategies island-wide, especially targeting *A. ceylanicum*, a zoonotic hookworm, that may establish patent infections in both companion animals and humans.

**Graphical Abstract:**

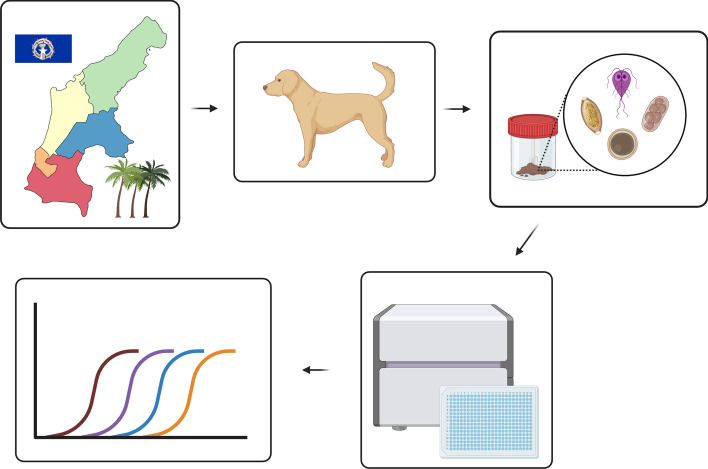

**Supplementary Information:**

The online version contains supplementary material available at 10.1186/s13071-026-07258-8.

## Background

Gastrointestinal (GI) parasites of dogs, including helminths and protozoans, are of substantial relevance to veterinary medicine and public health [1–7]. Among the most common GI parasites infecting dogs worldwide are nematodes *Ancylostoma* spp., *Toxocara canis*, and *Trichuris vulpis*; the flea tapeworm *Dipylidium caninum*; and protozoans such as *Giardia duodenalis*, *Cystoisospora* spp., and *Cryptosporidium* spp. [4, 8–14].

Among nematodes of zoonotic importance are species of *Ancylostoma*, which may cause cutaneous larva migrans or establish patent infections in humans [15–18], and species of *Toxocara*, which are responsible for visceral or ocular larva migrans in humans [19]. In addition, a subclinical condition termed covert toxocariasis has been linked to cognitive and developmental delays, and can also impact humans, disproportionately affecting infants, pregnant women, and those living in poverty [20–23].

Nevertheless, epidemiological data on zoonotic GI parasites in dogs are scarce in many parts of the world, especially in remote areas [24]. The emergence of novel technologies and diagnostic platforms enables comprehensive screening for multiple GI parasites [25]. In addition, some commercially available platforms allow for concomitant assessment of the zoonotic potential of *Giardia* (i.e. assemblages A and B) and of the presence of beta-tubulin mutations associated with resistance to benzimidazole drugs, specifically the F167Y [26] and Q134H polymorphisms in *Ancylostoma caninum*, which has been an emerging issue in North America [27–36] and Australia [37].

To date, there has been limited surveillance of GI parasites in Saipan and the nearby islands. Guam, a neighboring island, recently conducted a study that screened for tick-borne pathogens in dogs, cats, and wild pigs [38]. Several pathogens were found in the sampled dogs, including *Anaplasma phagocytophilum* (5.9%), *Anaplasma platys* (19.1%), *Babesia canis vogeli* (8.8%), and *Hepatozoon canis* (14.7%) [38]. Although this study [38] identified several tick-borne pathogens in Guam, there is a need for active surveillance among remote populations to assess the distribution of zoonotic pathogens and potential risk factors for infection. Therefore, the objective of the present study was to assess the prevalence of GI parasites in dogs from Saipan, Northern Mariana Islands, and evaluate risk factors associated with infections.

## Methods

### Study area

Saipan is the second-largest island within the Commonwealth of the Northern Mariana Islands (CNMI), with a total land area of 119 km^2^ (15°11′14″N, 145°44′49″E) [39]. The human population on Saipan was estimated at 43,385 in the 2020 Census [39]. The island has a tropical rainforest climate with slight seasonal temperature variation, with a wet and a dry season [40].

**Fig. 1:**
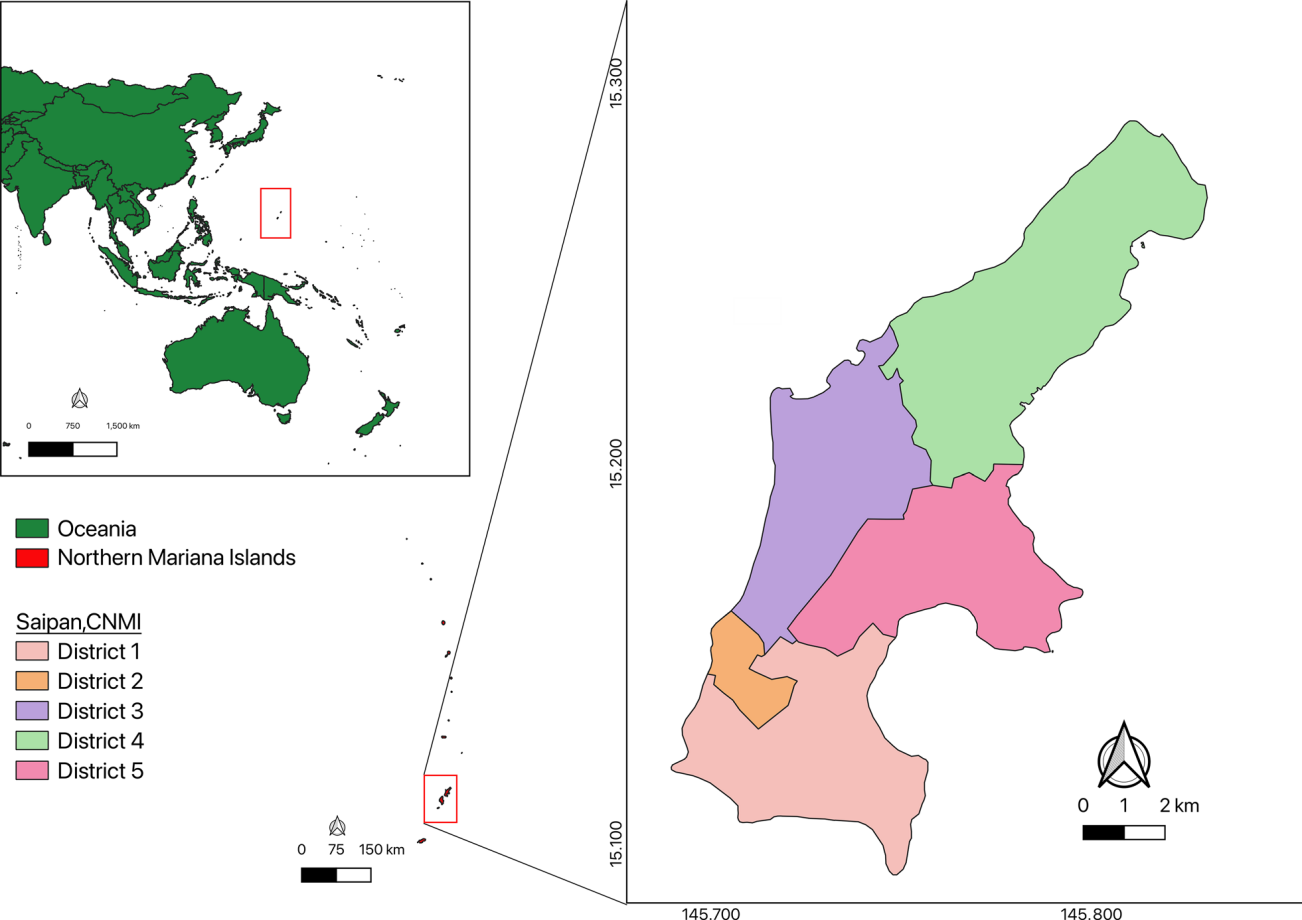
Aerial map of Saipan, Commonwealth of the Northern Mariana Islands, showing division of the five election districts, retrieved from the 115th Congress of the US Census Bureau, 2025

**Fig. 2:**
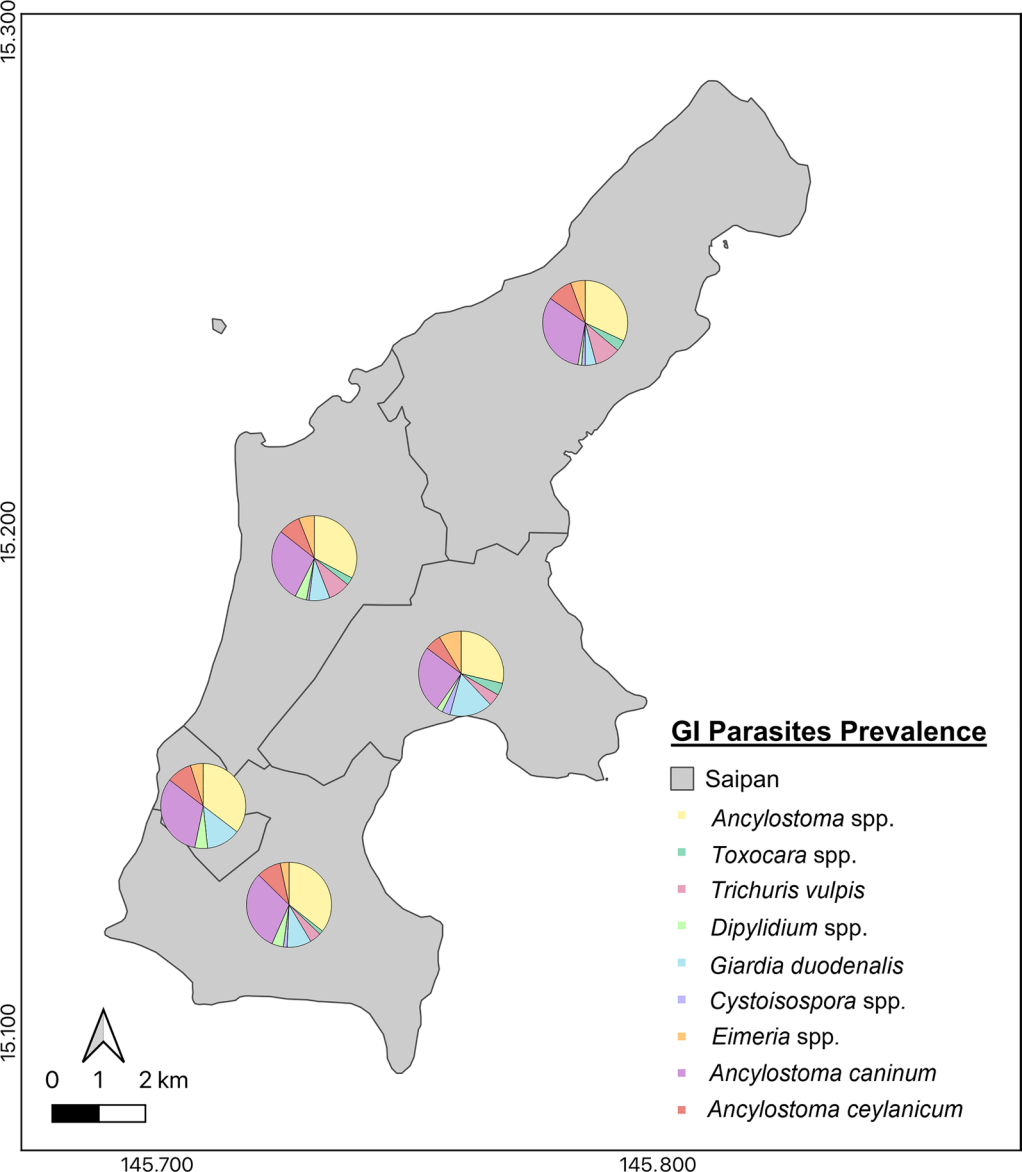
Prevalence of all gastrointestinal parasites detected within each resident district on Saipan, CNMI

All maps were created with QGIS 3.22.15, using the continuous cartographic base maps (Shapefiles, version 2018) from the United States (US) Census Bureau database, as shown in Fig. 1 [41]. The boundaries of districts 1–5 reflect the electoral districts on Saipan and are utilized for further assessment (Fig. 2).

### Sample collection

Canine fecal samples were collected using fecal loops or voluntarily voided feces as part of a broader study on canine health [42]. Briefly, demographics, including age, sex, residing location, and ownership status, were recorded and grouped accordingly for each dog. Age was separated into three groups: juveniles (≤ 1 year old), adults (1–7 years old), and seniors (> 7 years old), as reported or estimated by the owner or attending veterinarian, respectively. Each district was classified by the US Census Bureau as election districts 1–5 [41, 42]. Finally, ownership status was determined by the type of owner the dog had at the time of sampling, such as a client-owned dog, defined by whether the client wanted their dog to participate in the study. Owner-surrendered dogs were defined as those surrendered to the shelter within 2 days, whereas shelter dogs were defined as those living in the shelter for > 2 days, based on shelter records [42]. Samples were stored in individually labeled vials and kept frozen until shipment to TAMU. Following arrival at TAMU, samples were kept at – 20 °C until being shipped at 4 °C to Antech Diagnostics for processing.

### Sample processing and analysis

Total nucleic acid was extracted from each fecal sample according to the laboratory standard operating protocols at Antech Diagnostics. Briefly, following sample mixing, 150 mg of feces was combined with 750 µl of guanidinium thiocyanate-based lysis solution in a bashing bead tube (Spex Sample Prep, Metuchen, NJ, USA) and then homogenized according to the manufacturer’s recommendations. The cleared supernatant was used to extract total nucleic acids using a magnetic-bead-based automated extractor (KingFisher Apex, Thermo Fisher, Waltham, MA, USA). Nucleic acids were eluted in nuclease-free water and used for real-time PCR reactions. Subsequently, samples were screened with a commercially available GI parasite molecular test, which identifies 20 individual parasites, the F167Y and Q134H polymorphisms of *A. caninum* associated with resistance to benzimidazoles and *G. duodenalis* strains with zoonotic potential (KeyScreen™ GI Parasite PCR, Antech Diagnostics, Loveland, CO) [25, 26]. Real-time PCR tests in the gastrointestinal parasite panel were designed and validated according to established protocols [43]. DNA of the coccidian protozoan *Eimeria* spp. is targeted as an indicator of coprophagy and spurious parasite shedding. Additionally, two quality controls were used with each diagnostic sample, as previously described [34]. A complete list of parasites that can be detected by this assay and the associated gene targets can be found in Supplementary Data: Table 1. In addition to the qPCR tests included in the commercial intestinal parasite panel, a cytochrome b (*cytB*)-based qPCR test was developed to target feline genomic DNA in extracted stool samples as an indication for the presence of cat stool due to coprophagic behavior. This *cytB* gene feline specific test was designed against GenBank accession no. AB004238.1 and used for sequence alignments against canine, equine, bovine, caprine, ovine, and human *cytB* sequences. A region with low sequence identity between species was used to design a specific feline *cytB* gene qPCR test. Analytical validation confirmed the high specificity of the feline *cytB* gene qPCR (data not shown).

**Table 1:** Demographics of the dog population sampled from Saipan, CNMI (*n* = 420)

Demographics	No. (%)
Age	
Juvenile (≤ 1 year old)	102 (24.2)
Adult (> 1–7 years old)	264 (62.8)
Senior (> 7 years old)	54 (12.8)
Sex	
Female	244 (58.1)
Male	176 (41.9)
Residing location	
District 1	123 (29.2)
District 2	45 (10.7)
District 3	122 (29.0)
District 4	45 (10.7)
District 5	85 (20.2)
Ownership status	
Client-owned	318 (75.7)
Owner surrendered	48 (11.4)
Shelter	54 (12.8)

### Real-time PCR for *Ancylostoma* species co-detection

All samples that tested positive for *Ancylostoma* spp. through the KeyScreen™ GI Parasite PCR (Antech Diagnostics, Inc., Mars Petcare Science & Diagnostics, Loveland, CO, USA) test were further analyzed via qPCR targeting the ITS-1 gene to determine single and co-infections of *Ancylostoma* species. This *Ancylostoma* differentiation panel can distinguish *Ancylostoma caninum, A. braziliense*, and *A. tubaeforme* at the species level but does not differentiate *A. ceylanicum* and *A. duodenale*. Therefore, results are reported as *A. ceylanicum*/*A. duodenale*.

### Sequencing for *Ancylostoma* species determination

To allow species determination of those samples reported as *A. duodenale/A. ceylanicum*, genomic DNA was amplified with outside primers targeting the ITS-1 gene, and the species were determined using conventional Sanger sequencing. Briefly, PCR products were amplified using primers to obtain a product of between 236 to 262 bp depending on species. PCR products were purified with a proprietary magnetic bead-based 96-well purification protocol (Laguna Scientific, LLC., Aliso Viejo, CA, 92656). Purified PCR products were sequenced using conventional chain termination method using modified dideoxynucleotide triphosphates (ddNTPs) on an ABI 3730 (Applied Biosystems, Inc.). Obtained sequences were analyzed using the NCBI standard nucleotide BLASTn algorithms (http://ncbi.nlm.nich.gov/BLAST) with the function to align multiple sequence files. Following alignment, the 21 *A. ceylanicum* sequences showed 99–100% identity with four different reference sequences (PP527745; MG890213; MG589493, and KC755027) with lengths ranging between 236–262 bp. Thus, the *A. ceylanicum* sequence with the longest (258 bp) and 100% identity was the only sequence submitted to GenBank (accession no. PX279497).

### Data analysis

Statistical analysis was conducted using STATA version 19.5 BE-Basic Edition (Stata, College Station, TX, USA). Descriptive statistics were generated for each demographic variable and summarized in tables. Chi-square tests were performed to assess the association between potential risk factors—including age, sex, residing location, and dog ownership status—as exposures and each parasite as the outcome. A multivariable logistic regression was also performed for each parasite as the outcome, using a backward stepwise selection [44]. To evaluate how well each model fit the observed data, we used the Hosmer-Lemeshow and Pearson Χ^2^ tests [44]. We also generated both receiver-operating characteristic (ROC) curves and a ten-fold cross validation of ROC curves for each model to assess the performance [44]. Statistical significance was determined with a cutoff value of *P* ≤ 0.05. If perfect prediction occurred for specific model(s), the regression output was interpreted based on the reduced model and reported with caution [45, 46].

## Results

Of the sampled dogs, most were adults (> 1–7 years old; *n* = 264/420; 62.8%). Most of the dogs were female, accounting for 58.1% (*n* = 244/420) of the population, compared to 41.8% (*n* = 176/420) males. Samples were taken from all districts on the island, with the highest population in District 1, which comprised 29.2% (*n* = 123/420). Client-owned dogs (75.7%; *n* = 318/420) accounted for most of the ownership status demographic (Table 1).

Overall, parasites were detected in 267 (63.5%; 95% CI = 58.7–68.1) of canine samples (Table 2). The most detected parasite genus was *Ancylostoma* spp. (*n* = 224; 53.3%; 95% CI = 48.4–58.1), followed by *G. duodenalis* (*n* = 67; 15.9%; 95% CI = 12.5–19.8), *Trichuris* (*n* = 39; 9.2%; 95% CI = 6.6–12.4), *Dipylidium* (*n* = 25; 5.9%; 95% CI = 3.8–8.6), *Toxocara* (*n* = 15; 3.5%; 95% CI = 2.0–5.8), *Cystoisospora* (*n* = 10; 2.3%; 95% CI = 1.1–4.3), and *C. canis* (*n* = 5; 1.1%; 95% CI = 0.5–3.0). Assemblages with zoonotic potential of *G. duodenalis* and mutations conferring benzimidazole resistance in *A. caninum* were not detected using the methodology previously described [26]. *Eimeria*, used as a marker of spurious shedding, was found in 9.0% (*n* = 37; 95% CI = 0.5–3.0) of samples. Of the 37 dogs found to have *Eimeria* spp., 43% (*n* = 16/37) had co-infections with *Ancylostoma* spp., specifically *A. caninum* (*n* = 12/16) and *A. ceylanicum* (*n* = 2/16)*.* Two additional dogs were identified as co-infected with *A. caninum* and *A. ceylanicum* (*n* = 2/16). Results for all parasites detected in their respective districts are summarized in Fig. 2. Risk factors significantly associated with infection were age (*P* = 0.040), district (*P* = 0.006), and ownership (*P* =  < 0.001) for *Trichuris*; age and ownership for *Ancylostoma* (*P* =  < 0.001; *P* =  < 0.001), *G. duodenalis* (*P* =  < 0.001; *P* = 0.045), and *Dipylidium* (*P* = 0.011; *P* = 0.0.017), respectively; and ownership with *Toxocara* (*P* = 0.001) and *Cystoisospora* (*P* = 0.001) (Table 3).

**Table 2:** Prevalence for each gastrointestinal parasite detected in the sampled population using KeyScreen™ GI Parasite PCR

Parasite	No. positives	Prevalence (%)	Exact 95% CI
*Ancylostoma* spp.	144	34.2	29.7–39.0
*Eimeria* spp.	36	8.6	6.1–11.7
*Giardia duodenalis*	27	6.4	4.2–9.2
*Dipylidium* spp.	5	1.1	0.3–2.7
*Cystoisospora* spp.	3	0.4	0.1–2.0
*Trichuris* spp.	2	0.4	0.0–1.7
*Toxocara canis*	1	0.2	0.0–1.3
*Cryptosporidium canis*	1	0.2	0.0–1.3
Co-detections of two parasites			
*Ancylostoma* + *Trichuris*	21	5.0	3.1–7.5
*Ancylostoma* + *Giardia*	21	5.0	3.1–7.5
*Ancylostoma* + *Dipylidium*	9	2.1	0.9–4.0
*Ancylostoma* + *Cystoisospora*	6	1.4	0.5–3.0
*Ancylostoma* + *Toxocara*	4	0.9	0.2–2.4
*Giardia* + *Dipylidium*	4	0.9	0.2–2.4
*Ancylostoma* + *Cryptosporidium*	2	0.4	0.0–1.7
*Trichuris* + *Giardia*	1	0.2	0.0–1.3
Co-detections of three parasites			
*Ancylostoma* + *Trichuris* + *Giardia*	6	1.4	0.5–3.0
*Ancylostoma* + *Toxocara* + *Trichuris*	5	1.1	0.3–2.7
*Ancylostoma* + *Giardia* + *Dipylidium*	2	0.4	0.0–1.7
*Ancylostoma* + *Toxocara* + *Giardia*	1	0.2	0.0–1.3
*Ancylostoma* + *Trichuris* + *Dipylidium*	1	0.2	0.0–1.3
Co-detections of four parasites			
*Ancylostoma* + *Toxocara* + *Giardia* + *Dipylidium*	2	0.4	0.0–1.7
*Ancylostoma* + *Trichuris* + *Giardia* + *Dipylidium*	1	0.2	0.0–1.3
Co-detections of five parasites			
*Ancylostoma* + *Trichuris* + *Giardia* + *Dipylidium* + *Cryptosporidium*	1	0.2	0.0–1.3
*Ancylostoma* + *Trichuris* + *Giardia* + *Eimeria* + *Cryptosporidium*	1	0.2	0.0–1.3
*Toxocara* + *Trichuris* + *Giardia* + *Cystoisospora* + *Dipylidium*	1	0.2	0.0–1.3
Total parasites detected			
*Ancylostoma* spp.	224	53.3	48.4–58.1
*Giardia* spp.	67	15.9	12.5–19.8
*Trichuris* spp.	39	9.2	6.6–12.4
*Eimeria* spp.	37	8.8	6.3–11.9
*Dipylidium* spp.	25	5.9	3.8–8.6
*Toxocara canis*	15	3.5	2.0–5.8
*Cystoisospora* spp.	10	2.3	1.1–4.3
*Cryptosporidium canis*	5	1.1	0.3–2.7
Total	267	63.5	58.7–68.1

**Table 3:** Risk factors associated with prevalence of gastrointestinal parasites among dogs in Saipan

Variable	Total no.	*Ancylostoma* spp.			*Toxocara* spp.			*Trichuris* spp.			*Giardia duodenalis*			*Cystoisospora* spp.			*Dipylidium* spp.		
		No (%)	95% CI	Chi-square df *P*-value	No (%)	95% CI	Chi-square df *P*-value	No (%)	95% CI	Chi-square df *P*-value	No (%)	95% CI	Chi-square df *P*-value	No (%)	95% CI	Chi-square df *P*-value	No (%)	95% CI	Chi-square df *P*-value
Age																			
Juvenile	102	68 (66.7)	56.6–75.6	29.280 2 ** < 0.001**	5 (4.9)	1.6–11.0	4.434 2 0.109	13 (12.7)	6.9–20.8	6.447 2 **0.040**	29 (28.4)	19.9–38.2	15.564 2 ** < 0.001**	3 (2.9)	0.6–8.3	2.811 2 0.245	10 (9.8)	4.8–17.2	8.948 2 **0.011**
Adult	264	141 (53.4)	47.1–59.5		10 (3.7)	1.8–6.8		25 (9.4)	6.2–13.6		34 (12.8)	9.0–17.5		7 (2.6)	1.0–5.3		15 (5.6)	3.2–9.1	
Senior	54	12 (22.2)	12.0–35.5		0	–		1 (1.8)	0.0–9.8		4 (7.4)	2.0–17.8		0	–		0	–	
Sex																			
Female	244	123 (50.4)	43.9–56.8	1.141 1 0.285	6 (2.4)	0.9–5.2	2.057 1 0.151	26 (10.6)	7.0–15.2	1.326 1 0.249	40 (16.3)	11.9–21.6	0.084 1 0.771	8 (3.2)	1.4–6.3	2.208 1 0.137	15 (6.1)	3.4–9.9	0.039 1 0.842
Male	176	98 (55.6)	48.0–63.1		9 (5.1)	2.3–9.4		13 (7.3)	3.9–12.2		27 (15.3)	10.3–21.5		2 (1.1)	0.1–4.0		10 (5.6)	2.7–10.2	
Residing location																			
District One	123	73 (59.3)	50.1–68.1	5.465 4 0.243	3 (2.4)	0.5–6.9	5.368 4 0.252	9 (7.3)	3.4–13.4	14.451 4 **0.006**	19 (15.4)	9.5–23.0	8.766 4 0.067	3 (2.4)	0.5–6.9	4.044 4 0.400	9 (7.3)	3.4–13.4	3.323 4 0.505
District Two	45	22 (48.8)	33.7–64.2		0	–		0	–		8 (17.8)	8.0–32.0		0	–		3 (6.7)	1.3–18.2	
District Three	122	66 (54.1)	44.8–63.1		6 (4.9)	1.8–10.3		17 (13.9)	8.3–21.3		16 (13.1)	7.6–20.4		2 (1.6)	0.1–5.7		9 (7.3)	3.4–13.5	
District Four	45	23 (51.1)	35.7–66.2		3 (6.7)	1.3–18.2		7 (15.5)	6.4–29.4		3 (6.7)	1.3–18.2		1 (2.2)	0.0–11.7		1 (2.2)	0.0–11.7	
District Five	85	37 (43.5)	32.8–54.7		3 (3.5)	0.7–9.9		6 (7.0)	2.6–14.7		21 (24.7)	15.9–35.2		4 (4.7)	1.2–11.6		3 (3.5)	0.7–9.9	
Ownership																			
Client-owned	318	131 (41.1)	35.7–46.8	79.057 2 ** < 0.001**	5 (1.5)	0.5–3.6	13.879 2 **0.001**	8 (2.5)	1.0–4.8	60.892 2 ** < 0.001**	46 (14.4)	10.7–18.8	6.182 2 **0.045**	3 (0.9)	0.1–2.7	13.165 2 **0.001**	13 (4.0)	2.1–6.8	8.124 2 **0.017**
Owner-surrendered	48	45 (93.7)	82.8–98.6		3 (6.2)	1.3–17.1		12	13.6–39.5		14 (29.1)	16.9–44.0		1 (2.0)	0.0–11.0		4 (8.3)	2.3–19.9	
Shelter	54	45 (83.3)	70.7–92.0		7 (12.9)	5.3–24.9		19	22.6–49.3		7 (12.9)	5.3–24.9		6 (11.1)	4.1–22.6		8 (14.8)	6.6–27.1	

Multivariable logistic regression results for *Ancylostoma* spp. (Table 4) are summarized below. Juvenile dogs had 1.98 times higher odds (*P* = 0.013; 95% CI: 1.16—3.42) of having *Ancylostoma* spp. detected, whereas senior dogs had 65% lower odds (*P* = 0.005; 95% CI: 0.18—0.73) than adult dogs. Male dogs had 1.80 times higher odds of having *Ancylostoma* spp. detected (*P* = 0.010; 95% CI: 1.15—2.83) than female dogs. Owner-surrendered dogs had 18.22 times higher odds (*P* < 0.001; 95% CI: 5.33—62.30) and shelter dogs had 4.83 times higher odds (*P* < 0.001; 95% CI: 2.15—10.85) of having *Ancylostoma* spp. detected than client-owned dogs. Dogs found to have *Trichuris* had 3.99 times higher odds (*P* = 0.022; 95% CI: 1.23—13.04), whereas dogs found to have *G. duodenalis* had 51% lower odds (*P* = 0.030; 95% CI: 0.41—0.93) of having *Ancylostoma* spp. as a co-infection. Both the Hosmer-Lemeshow (*P* = 0.989) and Pearson chi-square (*P* = 0.231) tests were not significant, indicating no lack of fit [44]. We found that the area under the curve (AUC) for the ROC curve had fair accuracy (0.76) (Supplementary Data: Fig. 1) [45]. This was further confirmed in the ten-fold cross-validation AUC, which also showed fair accuracy (0.73) (Supplementary Data: Fig. 2) [47].

**Table 4:** Multivariable logistic regression for *Ancylostoma* species

*Ancylostoma* spp.	OR	SE	*Z*	*P*	95% CI
Age					
Adult	*	*	*	*	*
Juvenile	1.989	0.548	2.50	**0.013**	1.159—3.415
Senior	0.357	0.130	−2.83	**0.005**	0.175—0.729
Sex					
Female	*	*	*	*	*
Male	1.805	0.412	2.58	**0.010**	1.153—2.825
Ownership					
Client-owned	*	*	*	*	*
Owner-surrendered	18.227	11.430	1.63	** < 0.001**	5.332—62.302
Shelter	4.830	1.995	3.81	** < 0.001**	2.149—10.854
Co-infections					
*Trichuris* spp.	3.997	2.410	2.30	**0.022**	1.225—13.035
*Giardia duodenalis*	0.492	0.10	−2.18	**0.030**	0.260—0.931
Intercept	0.591	0.108	−2.86	0.004	0.413—0.847

Significant relationships (*p* < 0.05) denoted by bold font; *OR*: odds ratio; *SE*: standard error; *Z*: *Z* statistic; *P*: *P*-value; *CI*: confidence interval; *reference category

Juvenile dogs had 2.92 times higher odds (*P* = 0.001; 95% CI: 1.58—5.41) of having *G. duodenalis* detected than adult dogs. Owner-surrendered dogs had 2.83 times higher odds (*P* = 0.022; 95% CI: 1.16—6.89) of having *G. duodenalis* detected than client-owned dogs (Table 5). Dogs from district 5 had 2.50 times higher odds (*P* = 0.019; 95% CI: 1.16—5.41) than dogs of district 1 to have *G. duodenalis* detected. Dogs found to have *Dipylidium* had 4.19 times higher odds (*P* = 0.003; 95% CI: 1.64—10.73) of having *G. duodenalis* co-infection detected. We did not find evidence of lack of fit for the final model (Hosmer-Lemeshow *P* = 0.56; Pearson *χ*^2^ *P* = 0.81) [44]. We also found that the single AUC evaluation in the ROC curve had fair accuracy (0.74) (Supplementary Data: Fig. 3), but the ten-fold validation AUC showed poor accuracy (0.62) (Supplementary Data: Fig. 4) [47].

**Table 5:** Multivariable logistic regression for *Giardia duodenalis*

*Giardia duodenalis*	OR	SE	*Z*	*P*	95% CI
Age					
Adult	*	*	*	*	*
Juvenile	2.927	0.918	3.43	**0.001**	1.583—5.413
Senior	0.442	0.257	−1.40	0.162	0.141—1.385
Ownership					
Client-owned	*	*	*	*	*
Owner-surrendered	2.832	1.284	2.30	**0.022**	1.164—6.889
Shelter	0.695	0.378	−0.67	0.505	0.239—2.019
Residing district					
One	*	*	*	*	*
Five	2.508	0.985	2.34	**0.019**	1.161—5.416
Four	0.369	0.252	−1.46	0.145	0.096—1.411
Three	0.739	0.294	−0.76	0.449	0.338—1.614
Two	1.707	0.856	1.07	0.286	0.639—4.561
Co-infections					
*Ancylostoma* spp.	0.533	0.178	−1.87	0.061	0.277—1.029
*Dipylidium* spp.	4.198	2.010	3.00	**0.003**	1.642—10.730
*Eimeria* spp.	0.363	0.222	−1.65	0.099	0.109—1.207
*Trichuris* spp.	2.450	1.249	1.76	0.079	0.902—6.656
Intercept	0.137	0.048	−5.67	< 0.001	0.069—0.272

Significant relationships (*p* < 0.05) denoted by bold font; *OR*: odds ratio; *SE*: standard error; *Z*: *Z* statistic; *P*: *P*-value; *CI*: confidence interval; *reference category

The regressions for *Trichuris*, *Dipylidium*, *Toxocara canis*, *Cystoisospora*, and *Cystoisospora canis* are reported in the supplementary data. This is because each model demonstrated perfect prediction [45, 46]. To prevent misleading associations, we present the observed odds ratios from these specific models below, but we caution that these estimates are unreliable [45, 46].

Owner-surrendered dogs had 3.82 times higher odds (*P* < 0.001; 95% CI: 2.41—13.90), while shelter dogs had 13.41 times higher odds (*P* < 0.001; 95% CI: 5.09—35.34) of having *Trichuris* detected than client-owned dogs (Supplementary Data: Table 2). Dogs found to have *Ancylostoma* spp. had 3.25 times higher odds (*P* = 0.048; 95% CI: 1.00—10.47), whereas dogs found to have *Toxocara* had 4.16 times higher odds (*P* = 0.025; 95% CI: 1.19—14.48) of having *Trichuris* detected as a co-infection. We found no evidence of a lack of fit in the final model (Hosmer-Lemeshow *P* = 0.34; Pearson *χ*^2^ *P* = 0.38) [44]. Evaluating the ROC curve, the AUC had considerable accuracy (0.86) (Supplementary Data: Fig. 5), along with the ten-fold cross-validation AUC (0.80) (Supplementary Data: Fig. 6) [47].

Shelter dogs had 3.71 times higher odds (*P* = 0.007; 95% CI: 1.42—9.71) of having *Dipylidium* detected than client-owned dogs (Supplementary Data: Table 3). Dogs that were infected with *G. duodenalis* had 3.92 times higher odds (*P* = 0.002; 95% CI: 1.62—9.50) of having *Dipylidium* detected as a co-infection. There was no evidence of lack of fit in the final regression model (Hosmer-Lemeshow *P* = 0.97; Pearson *χ*^2^ *P* = 0.71) [44]. Following the single AUC evaluation in the initial ROC curve, we observed fair accuracy (0.70) for the final model [47] (Supplementary Data: Fig. 7). Additionally, the ten-fold cross-validation AUC showed a failure in accuracy (0.57) (Supplementary Data: Fig. 8) in the final model performance [47].

Dogs found to have both *Dipylidium* and *Trichuris* had 6.92 times (*P* = 0.012; 95% CI: 1.54—31.17) and 13.54 times higher odds (*P* < 0.001; 95% CI: 4.02—45.55), respectively, of having *T. canis* detected as a co-infection (Supplementary Data: Table 4). No evidence of lack of fit was found for the final model (Hosmer-Lemeshow *P* = 0.16; Pearson *χ*^2^ *P* = 0.11) [44]. The AUC for the initial ROC curve showed considerable accuracy (0.85) (Supplementary Data: Fig. 9), whereas the AUC for the ten-fold cross-validation had poor accuracy (0.62) (Supplementary Data: Fig. 10) [47].

Shelter dogs had 1.75 times higher odds of having *Cystoisospora* detected (*P* = 0.001; 95% CI: 2.47—42.62) than client-owned dogs (Supplementary Data: Table 5). Estimation of the final models' lack of fit was not possible, as neither the Hosmer-Lemeshow nor the Pearson chi-square tests yielded values that could be reported [44]. When evaluating the initial ROC, the AUC showed fair accuracy (0.65) (Supplementary Data: Fig. 11), whereas the ten-fold cross-validation failed (0.61) (Supplementary Data: Fig. 12) [47].

Dogs found to have *Trichuris* had 11.45 times higher odds (*P* = 0.010; 95% CI: 1.79—73.17) of being co-infected with *C. canis* (Supplementary Data: Table 6). Assessing the model's lack of fit was not possible because both the Hosmer-Lemeshow and Pearson chi-square tests yielded no values [44]. Area under the curve in the initial ROC curve presented poor accuracy (0.76) (Supplementary Data: Fig. 13) and was slightly lower in the ten-fold cross-validation (0.57) (Supplementary Data: Fig. 14) [47]. Hookworm-detected samples were further characterized to the species level using qPCR. Overall, *A. caninum* and *A. ceylanicum*/*A. duodenale* were confirmed in 196 (46.7%) and 57 (13.5%) dogs, respectively (Table 6). Further sequencing confirmed the presence of the zoonotic* A. ceylanicum* in at least 21 samples. The 21 dogs confirmed to have *A. ceylanicum* were geographically spread across all districts and accounted for about 5% of the sampled population. Samples containing *A. duodenale* species were not selected for Sanger sequencing; however, three dogs (0.7%) were found to have a single infection, and two dogs (0.4%) had a co-infection with *A. caninum*.

**Table 6:** *Ancylostoma* species specified between qPCR and cPCR

Single detections	KeyScreen™ species typing assay		Sanger sequencing	
	No. positive (%)	Exact 95% CI	No. positive (%)	Exact 95% CI
*A. caninum*	161 (38.3)	33.6–43.1	–	–
*A. duodenale/ceylanicum*	22 (5.2)	3.3–7.0	21 (5.0)	3.1–7.5
*A. tubaeforme*	3 (0.7)	0.1–2.0	–	–
Co-detections				
*A. caninum* + *A. duodenale/ceylanicum*	33 (7.8)	5.4–10.8	–	–
*A. caninum* + *A. tubaeforme*	2 (0.4)	0.0–1.0	–	–
*A. tubaeforme* + *A. duodenale/ceylanicum*	2 (0.4)	0.0–1.0	–	–
*A. caninum* + *A. duodenale*	–	–	3 (0.7)	0.1–2.0
*A. caninum* + *A. ceylanicum*	–	–	1 (0.2)	0.0–1.3
Sequencing failed	1	–	1	–
Total	224 (53.3)	48.4–58.1	26 (6.1)	4.0–8.9

Risk factors associated with *A. caninum* included age (*P* < 0.001) and ownership status (*P* < 0.001) (Table 7). Following the multivariable logistic regression, juvenile dogs had 2.46 times higher odds (*P* = 0.001; 95% CI: 1.44—4.21) of having *A. caninum* detected, whereas senior dogs had 60% lower odds (*P* = 0.019; 95% CI: 0.19—0.86) (Table 8). Male dogs had 1.77 times higher odds (P = 0.013; 95% CI: 1.12—2.78) of having *A. caninum* than female dogs. Owner-surrendered dogs had 10.26 times higher odds (*P* =  < 0.001; 95% CI: 4.19—25.08) of having *A. caninum* detected than shelter dogs having 6.54 times higher odds (*P* =  < 0.001; 95% CI: 2.95—14.50). Dogs found to have *G. duodenalis* had 60% lower odds (*P* = 0.007; 95% CI: 0.21—0.77) of being co-infected with *A. caninum*. We found no evidence of lack of fit (Hosmer-Lemeshow *P* = 0.6890; Pearson *χ*^2^ *P* = 0.8195) [44]. For this model, the single ROC curve AUC value was fair (0.76) (Supplementary Data: Fig. 15), as with the ten-fold cross-validation, which found similar accuracy (0.72) (Supplementary Data: Fig. 16) [47].

**Table 7:** Risk factors associated with *Ancylostoma caninum* (*n* = 196) in dogs

Variable	Total no.	*Ancylostoma caninum* (*n* = 196)		
		No. (%)	95% CI	Chi-square df *P*-value
Age				
Juvenile	102	62 (60.7)	50.6–70.3	26.994 2 ** < 0.001**
Adult	264	124 (46.9)	40.8–53.1	
Senior	54	10 (18.5)	9.2–31.4	
Sex				
Female	244	107 (43.8)	37.5–50.3	1.852 1 0.173
Male	176	89 (50.5)	42.9–58.1	
Residing location				
District 1	123	63 (51.2)	42.0–60.3	3.592 4 0.464
District 2	45	20 (44.4)	29.6–60.0	
District 3	122	57 (46.7)	37.6–55.9	
District 4	45	23 (51.1)	35.7–66.2	
District 5	85	33 (38.8)	28.4–50.0	
Ownership				
Client-owned	318	111 (34.9)	29.6–40.4	77.342 2 ** < 0.001**
Owner-surrendered	48	41 (85.4)	72.2–93.9	
Shelter	54	44 (81.4)	68.5–90.7

**Table 8:** Multivariable logistic regression for *Ancylostoma caninum*

*Ancylostoma caninum*	OR	SE	*Z*	*P*	95% CI
Age					
Adult	*	*	*	*	*
Juvenile	2.466	0.975	3.29	**0.001**	1.441—4.218
Senior	0.404	0.155	-2.35	**0.019**	0.189—0.861
Sex					
Female	*	*	*	*	*
Male	1.772	0.407	2.49	**0.013**	1.129—2.781
Ownership					
Client-owned	*	*	*	*	*
Owner-surrendered	10.262	4.678	5.11	** < 0.001**	4.199—25.080
Shelter	6.548	2.657	4.63	** < 0.001**	2.955—14.506
Co-infections					
*Trichuris* spp.	2.343	1.189	1.68	0.093	0.867—6.335
*Giardia duodenalis*	0.407	0.134	−2.72	**0.007**	0.213—0.778
Intercept	0.427	0.080	−4.49	< 0.001	0.295—0.619

Significant relationships (*p* < 0.05) denoted by bold font; *OR*: odds ratio; *SE*: standard error; *Z*: *Z* statistic; *P*: *P*-value; *CI*: confidence interval; *reference category

In Table 9, we found that *A. ceylanicum* had a statistically significant correlation with age (*P* = 0.030) and ownership (*P* = 0.002). In multivariable logistic regression, owner-surrendered dogs had 2.50 times higher odds (*P* = 0.016; 95% CI: 2.95—14.50) of having *A. ceylanicum* than client-owned dogs (Table 10). Dogs found to have *Trichuris* had 2.74 times higher odds of having *A. ceylanicum* detected as a co-infection. No significant evidence was found to suggest lack of fit (Hosmer-Lemeshow *P* = 0.7291; Pearson *χ*^2^ *P* = 0.1488) [44]. The initial ROC curve AUC had poor accuracy (0.63) (Supplementary Data: Fig. 17). Furthermore, the ten-fold cross-validation failed in accuracy (0.55) (Supplementary Data: Fig. 18) [47].

**Table 9:** Risk factors associated with *Ancylostoma ceylanicum* (*n* = 57)

Variable	Total no.	*Ancylostoma ceylanicum*		
		No. (%)	95% CI	Chi-square df *P*-value
Age				
Juvenile	102	17 (16.6)	10.0–25.3	6.984 2 **0.030**
Adult	264	38 (14.3)	10.3–19.2	
Senior	54	2 (3.7)	0.4–12.7	
Sex				
Female	244	38 (15.5)	11.2–20.7	2.032 1 0.154
Male	176	19 (10.8)	6.6–16.3	
Residing location				
District 1	123	19 (15.4)	9.5–23.0	1.899 4 0.754
District 2	45	6 (13.3)	5.0–26.7	
District 3	122	17 (13.9)	8.3–21.3	
District 4	45	7 (15.5)	6.4–29.4	
District 5	85	8 (9.4)	4.1–17.7	
Ownership				
Client-owned	318	32 (10.0)	6.9–13.9	12.658 2 **0.002**
Owner-surrendered	48	13 (27.0)	15.2–41.8	
Shelter	54	12 (22.2)	12.0–35.5

**Table 10:** Multivariable logistic regression for *Ancylostoma ceylanicum*

*Ancylostoma ceylanicum*	OR	SE	*Z*	*P*	95% CI
Ownership					
Client-owned	*	*	*	*	*
Owner-surrendered	2.507	0.969	2.38	**0.017**	1.174—5.351
Shelter	1.525	0.639	1.01	0.314	0.670—3.470
Co-infections					
*Trichuris* spp.	2.749	1.590	2.42	**0.016**	1.210—6.244
Intercept	0.123	0.021	−11.73	< 0.001	0.086—0.174

Significant relationships (*p* < 0.05) denoted by bold font; *OR*: odds ratio; *SE*: standard error; *Z*: *Z* statistic; *P*: *P*-value; *CI*: confidence interval; *reference category

## Discussion

A high prevalence of parasite detections and putative infections was observed in the canine population in this remote island. To the authors' knowledge, this is the first study assessing the prevalence of GI parasites in dogs from Saipan and the Commonwealth of the Northern Mariana Islands. The findings in this study provide a baseline for future research and help inform veterinary and public health authorities.

The most frequently detected parasites were hookworms of the genus *Ancylostoma*, which are clinically significant because high infection levels are linked to anemia and can be fatal [2, 6, 48, 49]. Various studies worldwide have determined that hookworms are often the most prevalent GI nematode infecting dogs [7, 8, 50–54]. A high prevalence of hookworms is often observed in tropical regions worldwide, where environmental conditions, including temperature, humidity, and soil type, may favor parasite development and transmission [1, 6, 29, 55, 56]. Regarding species, *A. caninum* was the most prevalent, although mutations associated with resistance to benzimidazole drug were not detected, as seen in the continental USA, Brazil, and Australia [25, 34, 37, 57]. This may be partially explained by several factors, including the island's location in the Pacific Ocean, limited availability of veterinary care, and the lack of routine use of anthelmintic drugs for prevention and control of infections, as also suggested by Kelly et al. regarding canine vector-borne diseases, especially canine heartworm, *Dirofilaria immitis* [42, 58]. Additional evidence of the absence of resistant mutations in *Ancylostoma* spp. can be seen by examining the stray or free-ranging dog population on the island of Saipan [59]. Although animal control agencies oversee this population, it is still estimated to exceed 21,000 dogs. When managed through spay-and-neuter programs or shelter euthanasia, it can lead to high turnover, which results in fewer dogs receiving treatment and possible resistance to prevention [59]. While the factors mentioned increase the risk, the movement of infected dogs from areas with resistance may still facilitate the introduction of *A. caninum* mutations into the island’s population.

Epidemiological studies on canine GI parasites often find *Giardia* to be the most prevalent protozoan, which can cause severe clinical signs in young animals [2, 60–64]. This was also observed in the multivariable logistic regression for *Giardia*, with a statistical association in juveniles and owner-surrendered dogs. It was also found that *Dipylidium* showed a statistically significant difference in co-infections with *Giardia* compared to all other pathogens identified in this study. Owners who recently surrendered their dogs may have done so because they were unable to manage their dogs' clinical signs, leading them to surrender the animals to a shelter. However, despite being a common cause of diarrhea in dogs, there is a growing debate on the relevance of subclinical *Giardia* infections in companion animals [2, 63–65]. Nevertheless, the zoonotic potential of specific genetic assemblages of *Giardia* requires attention, and veterinarians may opt to molecularly characterize canine infections to better inform and manage cases [25, 26, 34]. Regarding *Giardia* assemblages A (*G. duodenalis*) and B (*G. enterica*), both are relevant to zoonotic transmission and have been reported in human infections [66–69]. Since these assemblages were not detected in the studied dog population, there may be a lower risk to humans in contact with these dogs. Although not further genotyped, this population is most likely to carry *Giardia* assemblages commonly found in dogs, such as C (*G. canis*) and D (*G. lupus*) [70–74].

*Trichuris*, or whipworms, are potentially zoonotic GI nematodes that pose diagnostic challenges and have a controversial clinical significance [53, 75–77]. Standard diagnostic techniques used to identify *Trichuris*, in particular *T. vulpis*, include fecal flotations, such as double-centrifugal flotation, for dogs [37, 78–80]. However, egg shedding is not usually found in high numbers; therefore, infections may be missed in a clinical setting [78–80]. While the primers used in this study would have potentially detected *Trichuris* species infecting both dogs and cats, *T. vulpis* was most likely the species found in this population [26]. *Trichuris vulpis*, which can cause diarrhea and weight loss, is found in dogs of all ages but is significantly associated with adult (> 1 year old) and senior (< 7 years old) dogs [2, 53, 81]. The same outcome was observed in this study: adults (9.4%; *n* = 25/264) had a higher infection rate than juveniles (12.7%; *n* = 13/102) in the sampled population. The final regression model also found a statistical association between owner-surrendered and shelter dogs, as well as significance for co-infections with *Ancylostoma* spp. and *T. canis*. This is because these age classes exhibit increased resilience, higher infection intensity, and co-infections with other parasites, such as *A. caninum* [53, 79, 81]. However, there is always an increased potential for infection when an owner does not seek regular veterinary care, cannot afford the cost of pet care, or when administration of prevention is not logistically feasible in shelter settings [53, 79, 81].

*Dipylidium caninum* is a cestode distributed worldwide in companion animals that uses *Ctenocephalides* fleas or *Trichodectes canis* lice as intermediate hosts [2, 82]. Although not officially reported, fleas are known to infest dogs and cats on Saipan (K. Anderson, personal observation). Diagnosis of patent *D. caninum* infections is often challenging because of the intermittent shedding of proglottids [3, 5, 78]. More recently, molecular and coproantigen detections have proven more sensitive and reliable than classical methods [5, 83, 84]. These novel diagnostic techniques may yield more accurate prevalence estimates when implemented, alone or in conjunction, in epidemiological studies. Although the regression produced problematic results, we found that shelter dogs had a higher likelihood of infection with *Dipylidium*, as well as co-infections with *G. duodenalis*. While *Dipylidium* may not be of high zoonotic importance, reports of infection in infants are not uncommon [84–87]. Only a few reports worldwide, including in India and Russia, have focused on adult human cases of *D. caninum* infection [88, 89].

Unfortunately, we were unable to record a detailed clinical history or the frequency of anthelmintic drug use for the dogs enrolled in this study. However, it was unlikely that owner-surrendered and shelter dogs were receiving anthelmintic medication regularly or at all (K. Anderson, personal observation). For client-owned dogs, there has been intermittent use of various commercially available antiparasitic drugs that could have activity against selected endoparasites and ectoparasites (e.g. fluralaner, ivermectin; K. Anderson, personal observation). A future study could further investigate access to veterinary care and the use of antiparasitic products on dogs from Saipan.

Species of *Toxocara* are one of the most prevalent zoonotic helminths in veterinary and human medicine, with clinical manifestations differing between visceral larva migrans (VLM) and ocular larva migrans (OLM) [2, 19, 93–100]. *Toxocara canis* and *T. cati* eggs are shed in the feces of free-roaming dogs and cats and hence contaminate soil, highlighting the need for surveillance to understand transmission among dogs, cats, and humans [2, 19, 20, 93–100]. In the present study, *Toxocara* had a statistically significant association with age (*P* = 0.040). Upon closer examination, prevalence was higher in adults (> 1–7 years old) than in juveniles (≤ 1 year old) and senior dogs (> 7 years old). Statistical evidence also found an association between *Dipylidium* and *Trichuris* as co-infections, highlighting the need for further surveillance of these three pathogens, especially in younger animals. However, this overall positivity may be an underestimation, as we did not sample puppies younger than 6 months, and the regression was problematic in evaluating these risk factors due to the low positivity (*n* = 15).

Other commonly reported protozoan parasites in this study include species of *Cystoisospora* and *Cryptosporidium*, which are of little to moderate clinical significance in healthy, immunocompetent dogs [8, 51, 53, 64, 74, 101]. Young age is often a factor associated with the presence of clinical infections for both protozoans in dogs, as observed with other gastrointestinal nematodes [8, 102]. Age class, among others, could not be accurately assessed in either pathogen regression model, likely because of the low positivity rates, and requires further surveillance to fully elucidate significant risk factors. Another limitation in evaluating age as a possible risk factor for underestimating the prevalence of both *Cystoisospora* and *Cryptosporidium* was the exclusion of puppies younger than 6 months.

As mentioned earlier, several multivariable logistic regression models exhibited perfect prediction, meaning that all observations with a specific covariate pattern tested either positive or negative for pathogens that had a low number of positive cases (≤ 39 positive dogs, e.g. *Trichuris*, *Dipylidium*, *T. canis*, *Cystoisospora*, and *C. canis*) [45, 46]. Under these conditions, the logistic regression algorithm automatically excluded perfectly predicted observations from model fitting, reducing the adequate sample size and limiting the estimation of adjusted effects for these pathogens [45, 46]. The reason for perfect prediction is likely the combination of rare pathogens and small cell counts within specific covariate strata rather than a statistical association [45, 46]. Therefore, these results should be interpreted with caution, and future research should reexamine these potential risk factors and their association with GI parasites.

Hookworm-detected samples were further characterized at the species level. Overall, *A. caninum* was confirmed in most samples (*n* = 196/225), with 161 of these being single-species infections. *Ancylostoma duodenale/ceylanicum* was found in in 57, including 26 single infections, and *A. tubaeforme* 7 samples. Through a qPCR assay, co-detection of hookworm species was found in 37 samples, with *A. caninum* + *A. duodenale/ceylanicum* (*n* = 33/37), *A. caninum* + *A. tubaeforme* (*n* = 2/37), and *A. duodenale/ceylanicum* + *A. tubaeforme* (*n* = 2/37). Age is a well-documented risk factor for *A. caninum*, with higher infection rates reported in younger animals [53–55]. We also identified a significant association between *A. caninum* and other GI parasites (i.e. *G. duodenalis*) in co-infections, suggesting that the initial infections occurred simultaneously [50–52]. An assay targeting the *cytB* gene in cats detected feline DNA in four of the seven samples containing *A. tubaeforme* DNA, probably indicating coprophagia and spurious parasitism [26, 43]. However, other studies have documented *A. tubaeforme* DNA in dogs, although they are not suitable hosts, which suggests that further studies may be needed [103].

*Ancylostoma ceylanicum* is of particular interest because it can establish patent infections in dogs, cats, and humans, with the possibility of cross-species transmission [48, 57, 104, 105]. The presence of this zoonotic parasite was confirmed via sequencing in at least 21 samples, or approximately 5% of the dogs sampled on this remote island. Although age class was not identified in the multivariable logistic regression, age has been documented to be a risk factor for puppies up to senior dogs, requiring further investigation in similar populations to determine a more conclusive exposure factor [104, 105]. A statistical association was observed between client-owned and owner-surrendered dogs, suggesting that the lack of ownership can directly increase exposure to newly emerging pathogens. To our knowledge, our study provides the first report of *A. ceylanicum* from the Northern Mariana Islands, and possibly the USA minor territories in Oceania. This finding is expected, given Saipan's geographic location relative to other known endemic areas for *A. ceylanicum* [105]. This species has been widely reported from various countries across South and Southeast Asia and Oceania, with variable prevalence across all three susceptible host species [17, 105–109]. This suggests that the infection could have originated in Asia, potentially because of tourism and relocation to this remote island [17, 105–110]. As *A. ceylanicum* can also cause patent infections in humans, additional testing is required in special cases associated with enteritis and diarrhea in both the resident population and visitors (e.g. tourists and military personnel) [107, 110, 111].

Although most GI parasites found in this canine population are nearly globally distributed, *A. ceylanicum* has never been reported in companion animals or humans in the continental USA, and, until recently, it had not been detected across the Americas. Recent studies have shown the presence of *A. ceylanicum* in dogs on other remote islands, such as Kiribati, an island in the central Pacific [106, 112], and Grenada, an island in the Caribbean [113, 114], and in humans from Ecuador, South America [115]. With this confirmation of *A. ceylanicum*, there is potential movement of companion animals, including dogs and cats, to the continental USA from Saipan and the Northern Mariana Islands. This movement can include client-owned animals, as well as service, military, and TSA dogs, given the island's military base, where reports of other parasitic and vector-borne infections have been reported in similar populations [116–120]. Currently, importation of dogs to the continental USA requires only four documents: the Centers for Disease Control and Prevention (CDC) Dog Import Form, a microchip confirmation certificate, a rabies vaccination certificate, and a health certificate [121]. However, records may be lost during travel to new locations or may not be followed closely upon arrival from outlying US territories, including the CNMI or Guam. Given the risk of translocation and establishment of parasites associated with dog importation, implementing more comprehensive parasite diagnostic testing prior to travel and a follow-up veterinary consultation could be informative to veterinary and public health professionals.

## Conclusions

To our knowledge, this is the first study providing epidemiological data on canine gastrointestinal parasites in Saipan. The high prevalence of infections and co-infections, such as *Ancylostoma* spp. and *Trichuris* spp., demonstrates that active surveillance to assess risk factors is crucial for understanding pathogen distribution in remote populations like Saipan. The results highlight the presence of parasites of veterinary and medical significance and emphasize the need for improved access to veterinary care, effective prevention measures, and the implementation of control strategies.

## Supplementary Information

Additional file1 (DOCX 2789 KB)

## Data Availability

All data analyzed during this study are included in this published article and its supplementary information files. The single submitted *Ancylostoma ceylanicum* sequence is available through the National Center for Biotechnology Information (NCBI) GenBank Database under accession no. PX279497.
