# Supplementary material for: High prevalence of gastrointestinal parasites in dogs from Saipan, Northern Mariana Islands, including the zoonotic Ancylostoma ceylanicum
Source: Parasit Vectors. 2026 Jan 28;19:93. doi: 10.1186/s13071-026-07258-8 (PMC12924339; doi:10.1186/s13071-026-07258-8)

**Supplementary Data, Table 1.** All parasites with associated gene targets capable of detection through the KeyScreen™ GI Parasite PCR Assay.

| **Parasite(s)** | **Gene Targets** |
| --- | --- |
| **Hookworms** | |
| *Ancylostoma* spp., *Ancylostoma caninum, Ancylostoma duodenale, Ancylostoma ceylanicum, Ancylostoma braziliense, Ancylostoma tubaeforme, Ancylostoma* markers associated with benzimidazole resistance, *Uncinaria stenocephala* | Ribosomal RNA genes, isotype-1 β-tubulin |
| **Roundworms** | |
| *Toxocara* spp., *Toxocara canis, Toxocara cati, Toxascaris leonina, Baylisascaris procyonis* | Ribosomal RNA gene |
| **Whipworms** | |
| *Trichuris vulpis, T. campanula, T. felis, T. serrata* | Ribosomal RNA gene |
| **Tapeworms** | |
| *Dipylidium caninum, Echinococcus multilocularis, Echinococcus granulosus, Taenia* spp. | Ribosomal RNA gene |
| ***Giardia*** | |
| *Giardia duodenalis, Giardia* assemblages A or B with zoonotic potential | Ribosomal RNA gene |
| **Coccidia (Sarcocystidae, Eimeridae)** | |
| *Cystoisospora canis, C. ohioensis complex, C. felis, C. rivolta, Eimeria* spp. | Ribosomal RNA gene |
| **Additional Protozoa** | |
| *Cryptosporidium canis, Cryptosporidium felis* | Ribosomal RNA gene |
| *Tritrichomonas blagburni, Neospora caninum, Toxoplasma gondii* |  |

**Supplementary Table 2**. Multivariable logistic regression for *Trichuris* species.

| *Trichuris* spp. | OR | SE | *Z* | *P* | 95% CI |
| --- | --- | --- | --- | --- | --- |
| Ownership | | | | | |
| Client-owned | * | * | * | * | * |
| Owner-surrendered | 3.826 | 3.621 | 3.62 | **<0.001** | 2.413—19.309 |
| Shelter | 13.417 | 6.631 | 5.25 | **<0.001** | 5.093—35.347 |
| Co-infections | | | | | |
| *Ancylostoma* spp. | 3.251 | 1.940 | 1.98 | **0.048** | 1.009—10.471 |
| *Cryptosporidium canis* | 7.825 | 8.583 | 1.88 | 0.061 | 0.911—67.166 |
| *Toxocara canis* | 4.163 | 2.648 | 2.24 | **0.025** | 1.196—14.482 |
| Intercept | 0.013 | 0.007 | -7.70 | <0.001 | 0.004—0.040 |

Significant relationships (*p*<0.05) denoted by bold font; *OR*: odds ratio; *SE*: standard error; *Z*: *Z* statistic; *P*: *P*-value; *CI*: confidence interval; *reference category

**Supplementary Table 3.** Multivariable logistic regression for *Dipylidium* species.

| *Dipylidium* spp. | OR | SE | *Z* | *P* | 95% CI |
| --- | --- | --- | --- | --- | --- |
| Ownership | | | | | |
| Client-owned | * | * | * | * | * |
| Owner-surrendered | 1.412 | 0.862 | 0.57 | 0.572 | 0.427—4.671 |
| Shelter | 3.715 | 1.821 | 2.68 | **0.007** | 1.421—9.712 |
| Co-infections | | | | | |
| *Giardia duodenalis* | 3.927 | 1.771 | 3.03 | **0.002** | 1.622—9.509 |
| Intercept | 0.036 | 0.012 | -9.91 | <0.001 | 0.018—0.069 |

Significant relationships (*p*<0.05) denoted by bold font; *OR*: odds ratio; *SE*: standard error; *Z*: *Z* statistic; *P*: *P*-value; *CI*: confidence interval; *reference category

**Supplementary Table 4**. Multivariable logistic regression for *Toxocara canis.*

| *Toxocara canis* | OR | SE | *Z* | *P* | 95% CI |
| --- | --- | --- | --- | --- | --- |
| Sex | | | | | |
| Female | * | * | * | * | * |
| Male | 3.208 | 1.952 | 1.92 | 0.055 | 0.973—10.573 |
| Co-infections | | | | | |
| *Dipylidium* spp. | 6.929 | 5.317 | 2.52 | **0.012** | 1.540—31.177 |
| *Trichuris* spp. | 13.545 | 8.382 | 4.21 | **<0.001** | 4.027—45.553 |
| Intercept | 0.009 | 0.006 | -7.48 | <0.001 | 0.002—0.033 |

Significant relationships (*P*<0.05) denoted by bold font; *OR*: odds ratio; *SE*: standard error; *Z*: *Z* statistic; *P*: *P*-value; *CI*: confidence interval; *reference category

**Supplementary Table 5**. Multivariable logistic regression for *Cystoisospora* species.

| *Cystoisospora* spp. | OR | SE | *Z* | *P* | 95% CI |
| --- | --- | --- | --- | --- | --- |
| Ownership | | | | | |
| Client-owned | * | * | * | * | * |
| Owner-surrendered | 1.751 | 2.043 | 0.48 | 0.631 | 0.178—17.240 |
| Shelter | 10.272 | 7.458 | 3.21 | **0.001** | 2.475—42.628 |
| Intercept | 0.013 | 0.007 | -1.44 | <0.001 | 0.004—0.041 |

Significant relationships (*p*<0.05) denoted by bold font; *OR*: odds ratio; *SE*: standard error; *Z*: *Z* statistic; *P*: *P*-value; *CI*: confidence interval; *reference category

**Supplementary Table 6**. Multivariable logistic regression for *Cryptosporidium canis.*

| *Cryptosporidium canis* | OR | SE | *Z* | *P* | 95% CI |
| --- | --- | --- | --- | --- | --- |
| Co-infections | | | | | |
| *Trichuris* spp. | 11.450 | 10.836 | 2.58 | **0.010** | 1.791—73.177 |
| Intercept | 0.010 | 0.005 | -7.89 | <0.001 | 0.003—0.032 |

Significant relationships (*p*<0.05) denoted by bold font; *OR*: odds ratio; *SE*: standard error; *Z*: *Z* statistic; *P*: *P*-value; *CI*: confidence interval; *reference category

**Supplementary Figure 1**. *Ancylostoma* species initial Receiving Operating Characteristic curve.


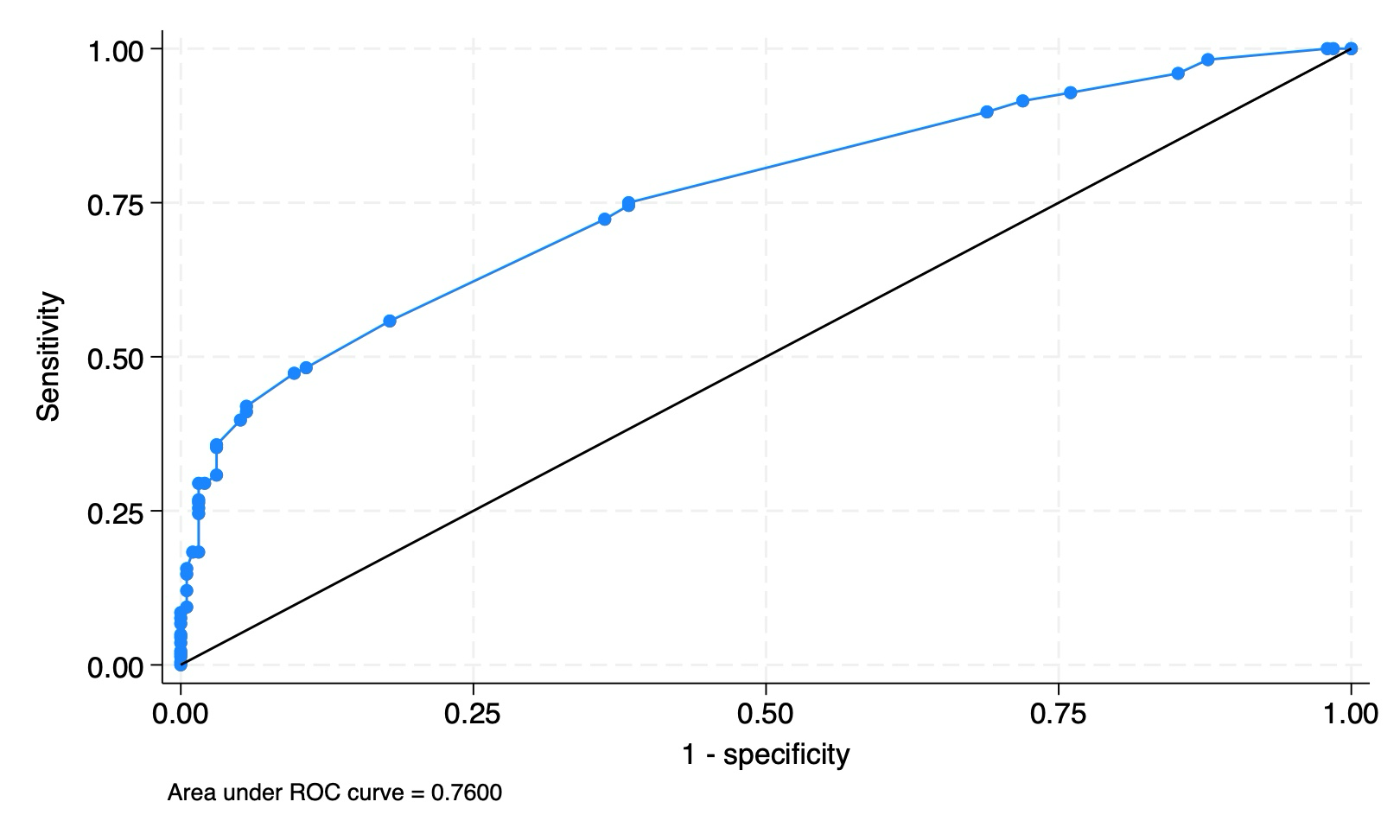


**Supplementary Figure 2**. *Ancylostoma* species ten-fold cross validation curve.


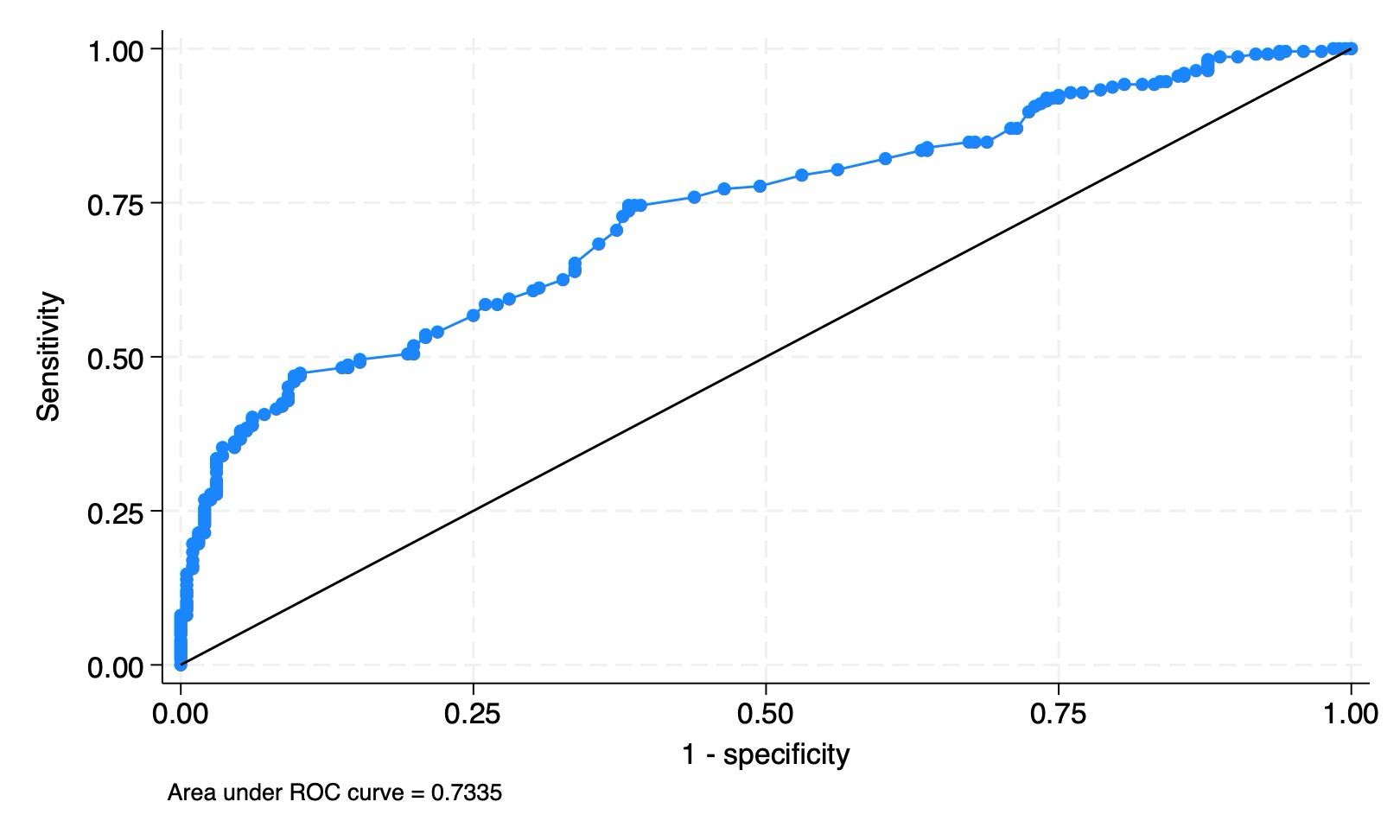


**Supplementary Figure 3**. *Giardia duodenalis* initial Receiving Operating Characteristic curve.


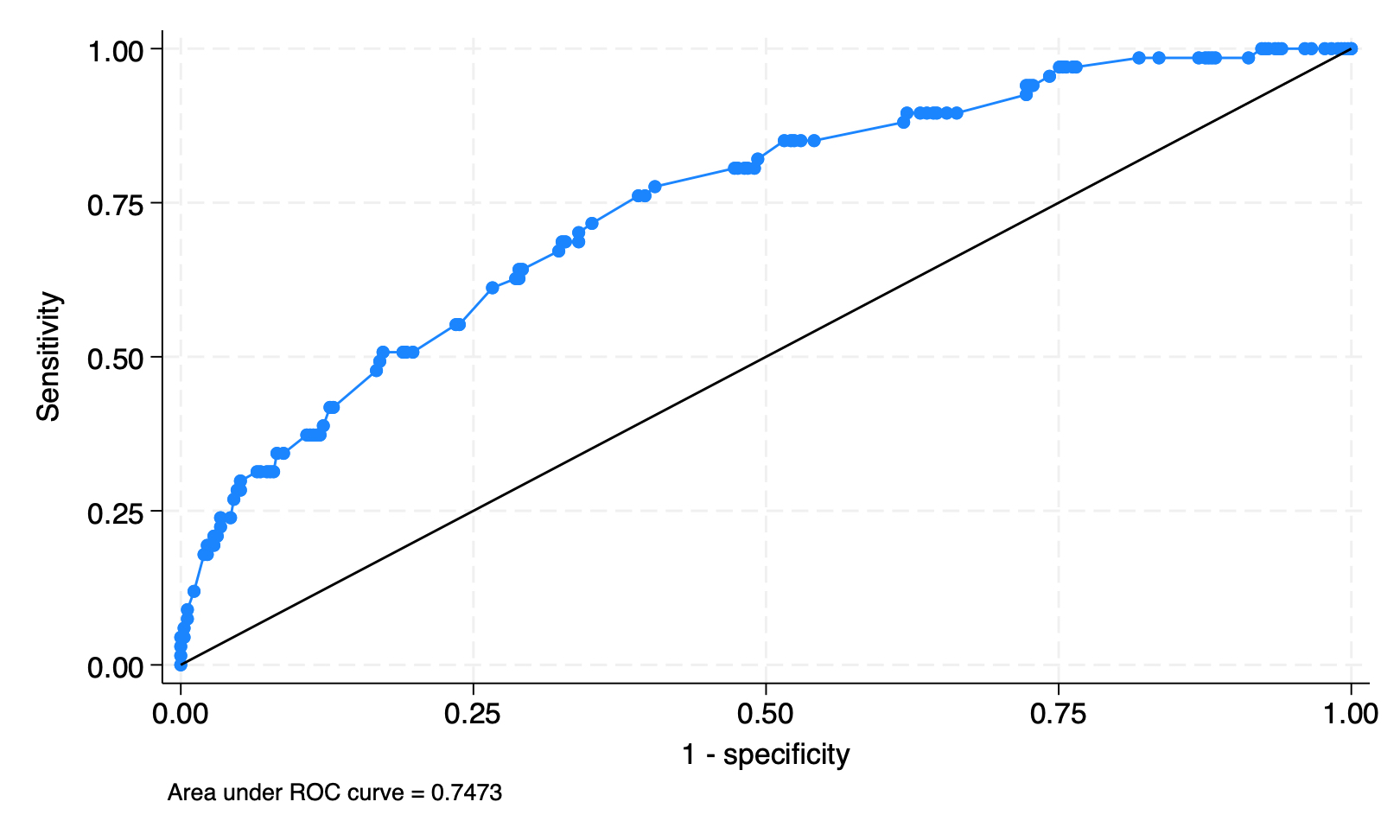


**Supplementary Figure 4**. *Giardia duodenalis* ten-fold cross validation curve.


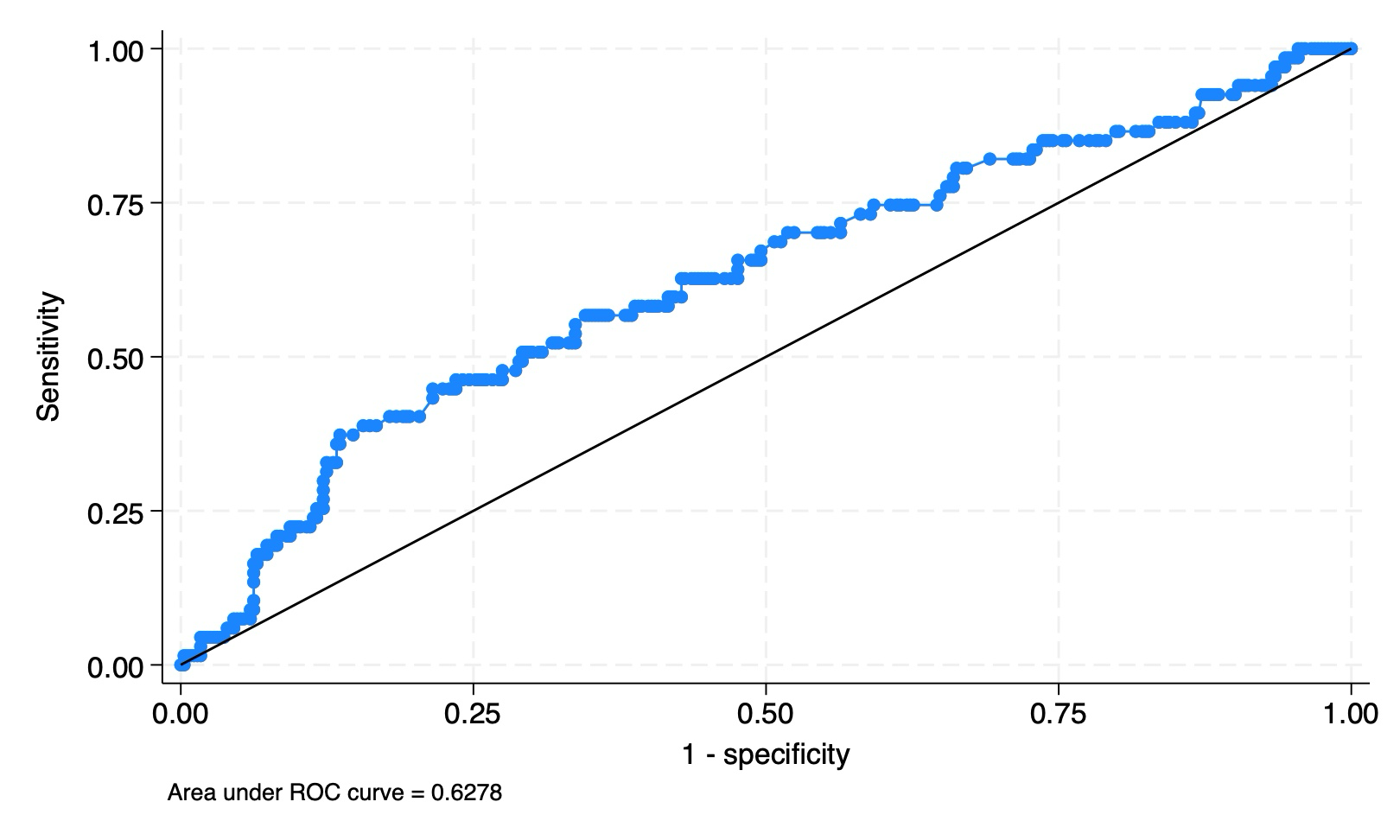


**Supplementary Figure 5**. *Trichuris* species initial Receiving Operating Characteristic curve.


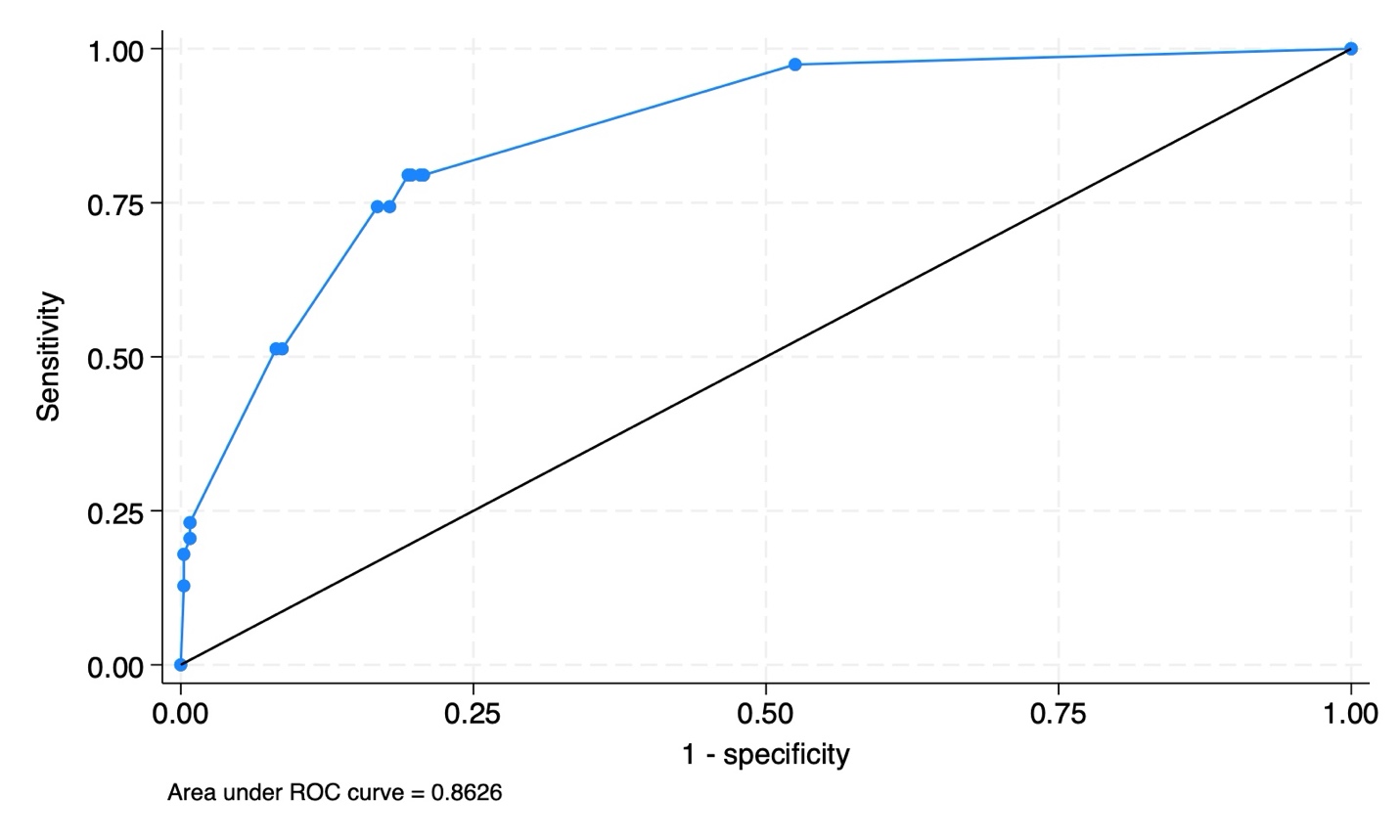


**Supplementary Figure 6**. *Trichuris* species ten-fold cross validation curve.


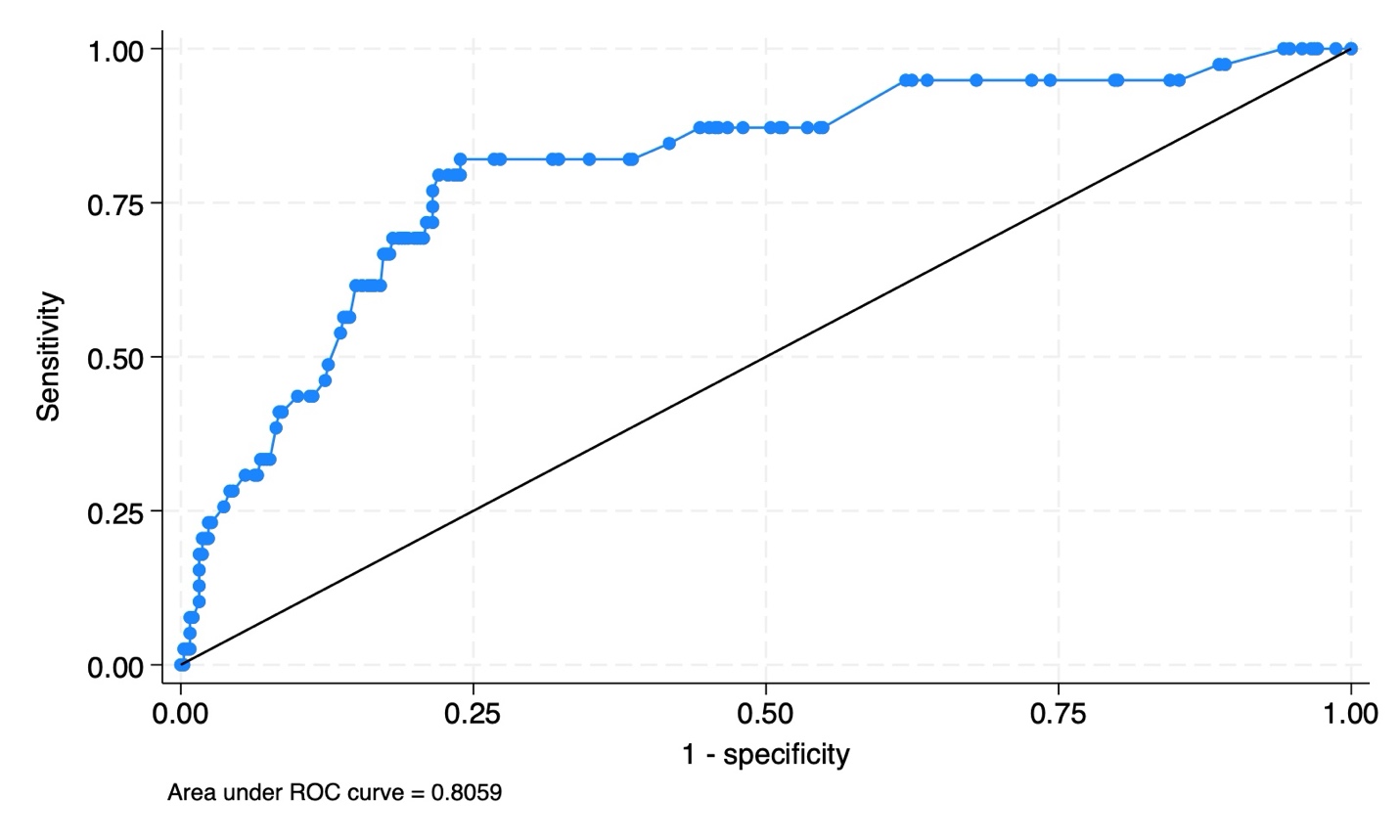


**Supplementary Figure 7**. *Dipylidium* species initial Receiving Operating Characteristic curve.


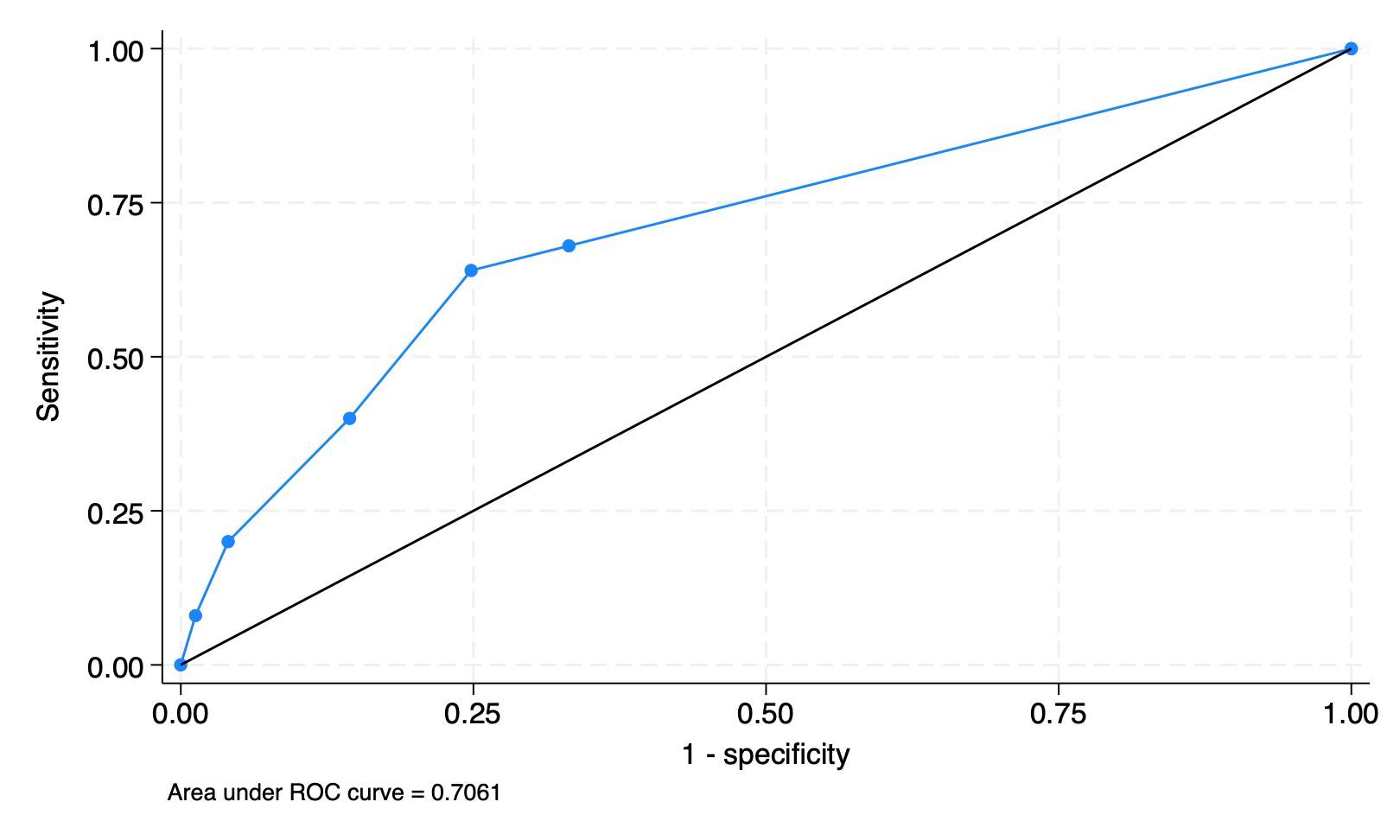


**Supplementary Figure 8**. *Dipylidium* species ten-fold cross validation curve.
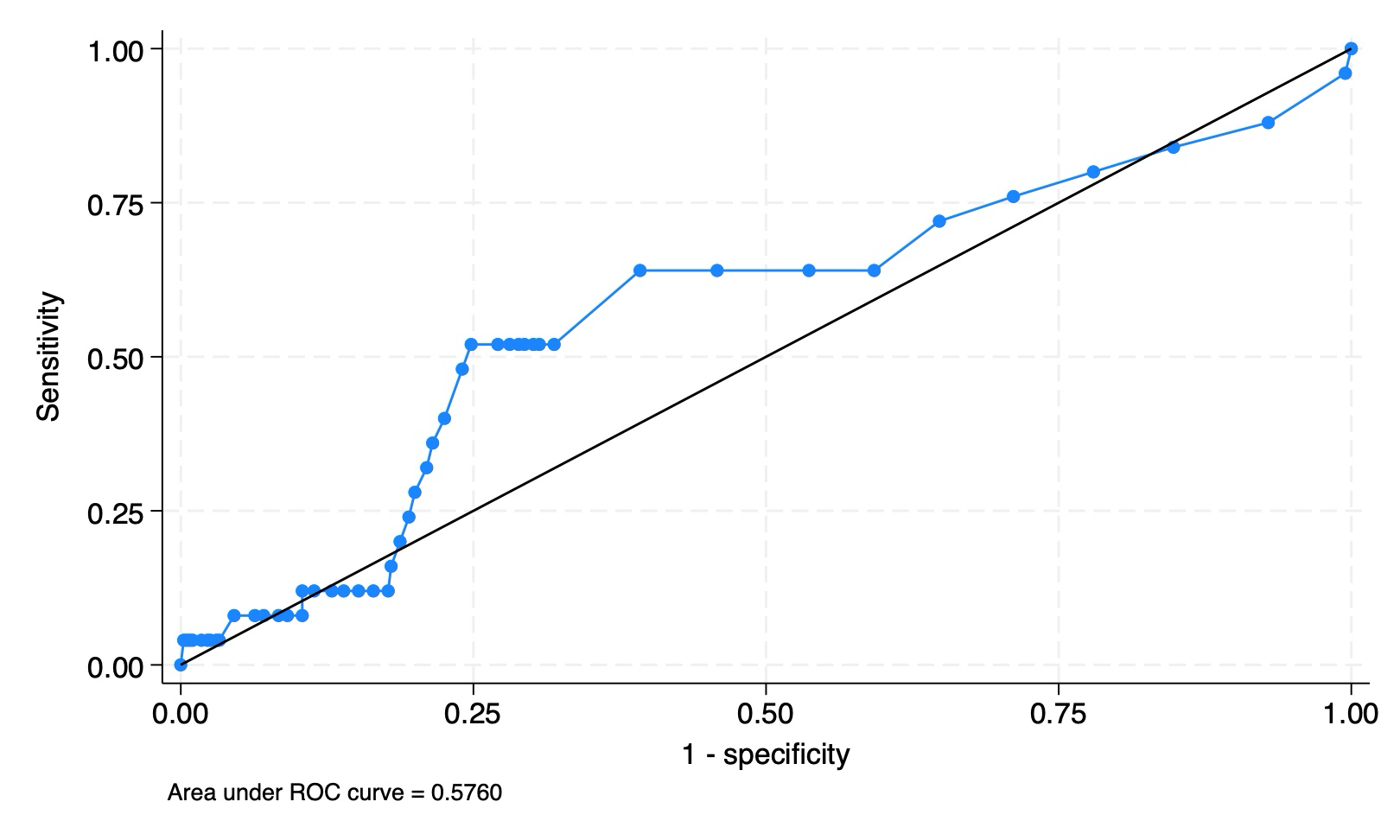


**Supplementary Figure 9**. *Toxocara canis* initial Receiving Operating Characteristic curve.


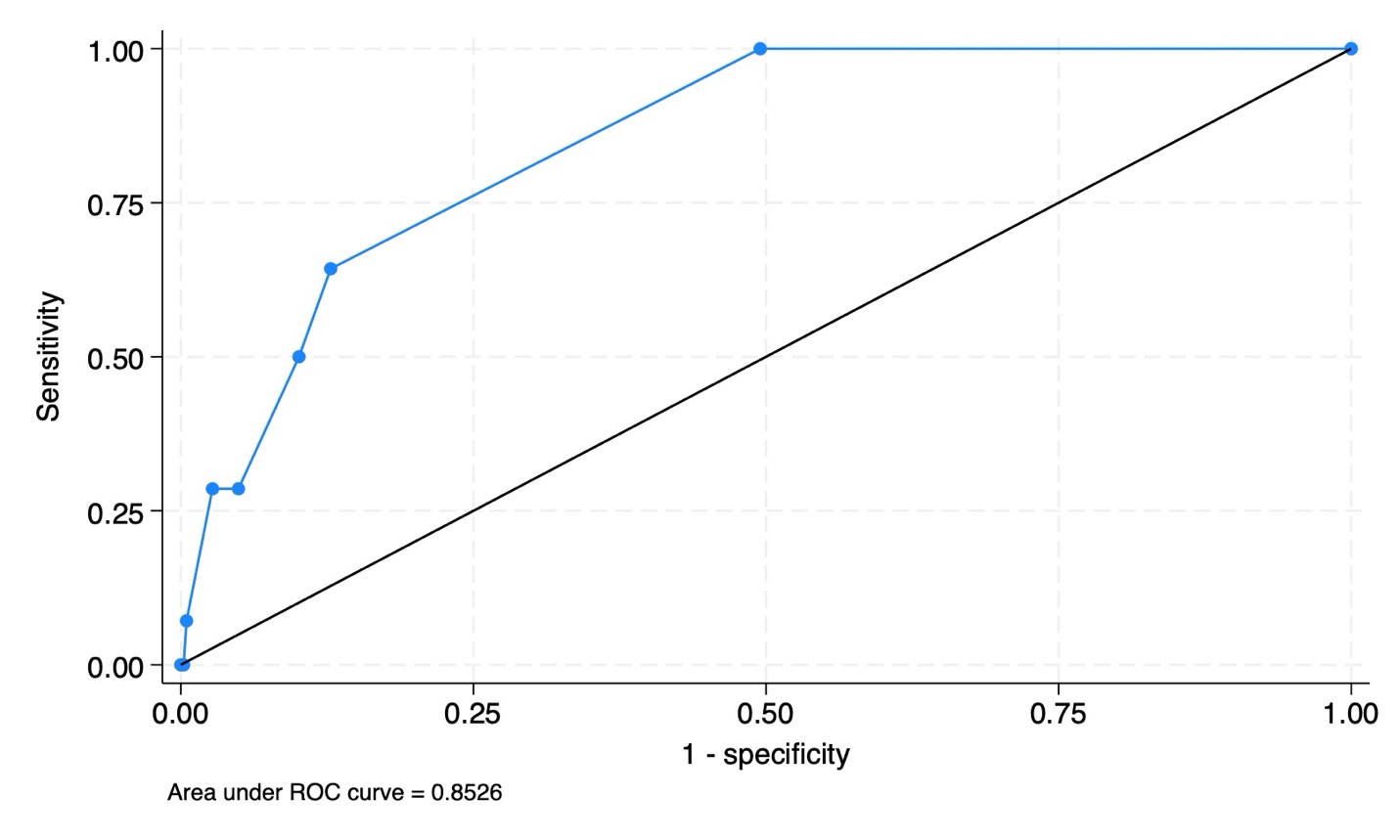


**Supplementary Figure 10**. *Toxocara canis* ten-fold cross validation curve.


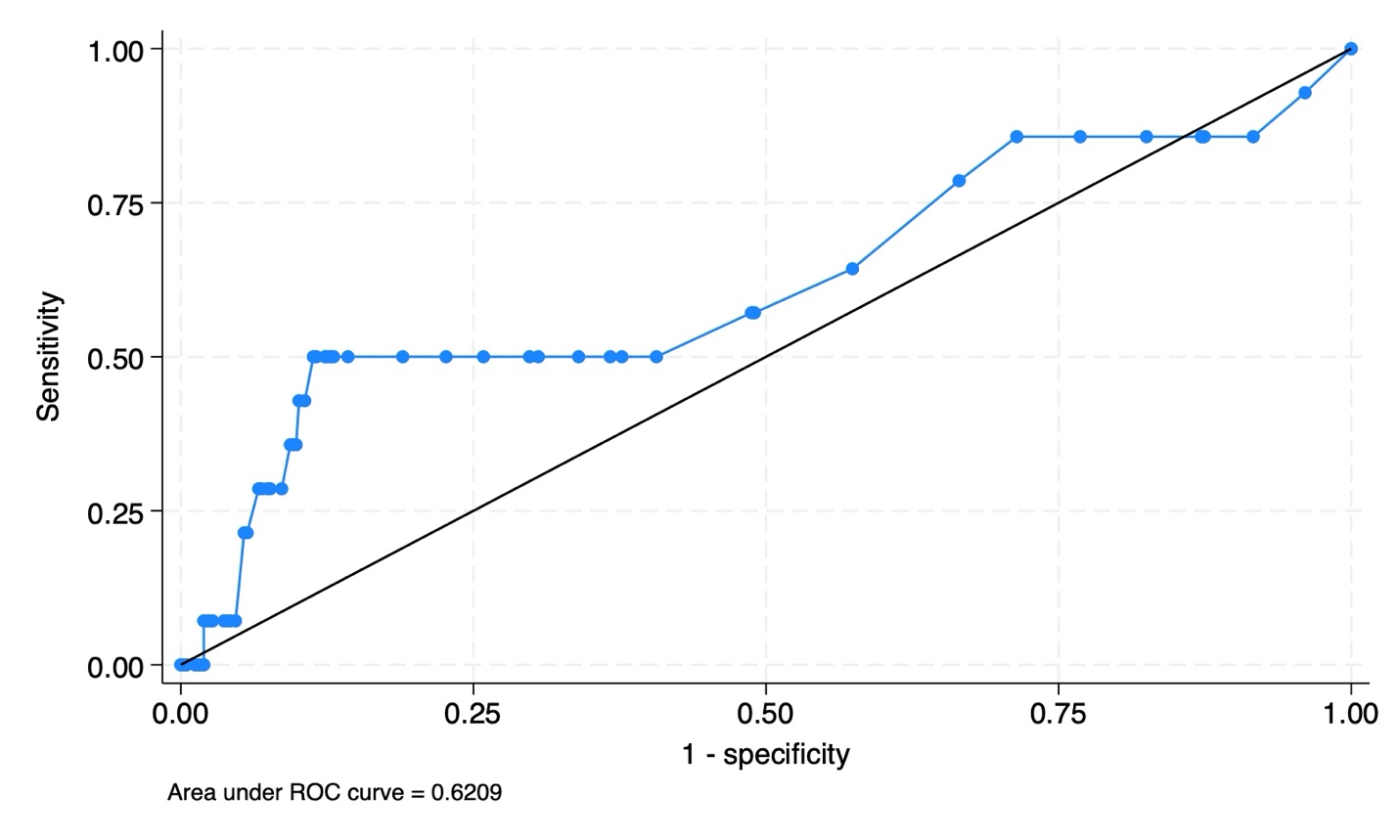


**Supplementary Figure 11**. *Cystoisospora* species initial Receiving Operating Characteristic curve.


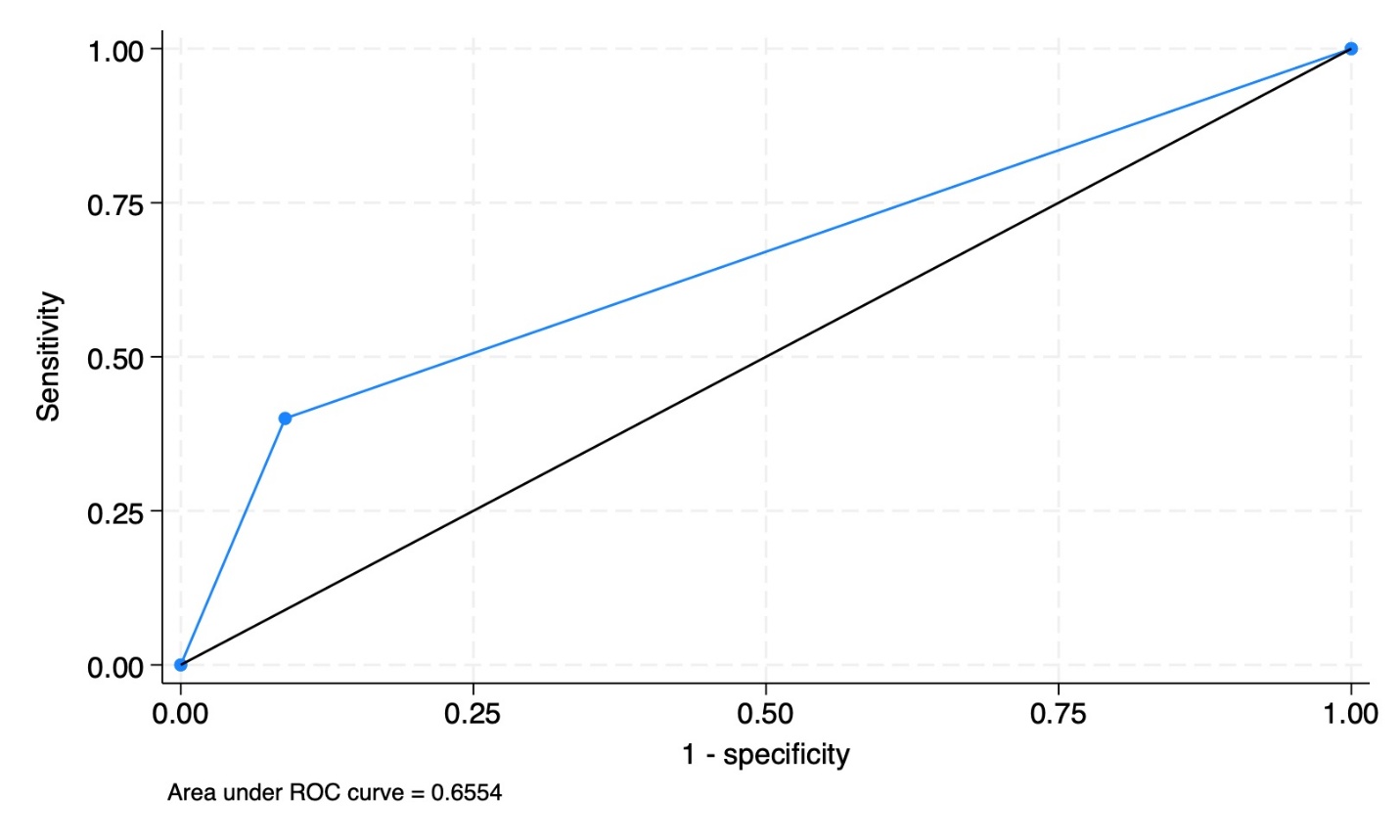


**Supplementary Figure 12**. *Cystoisospora* species ten-fold cross validation curve.


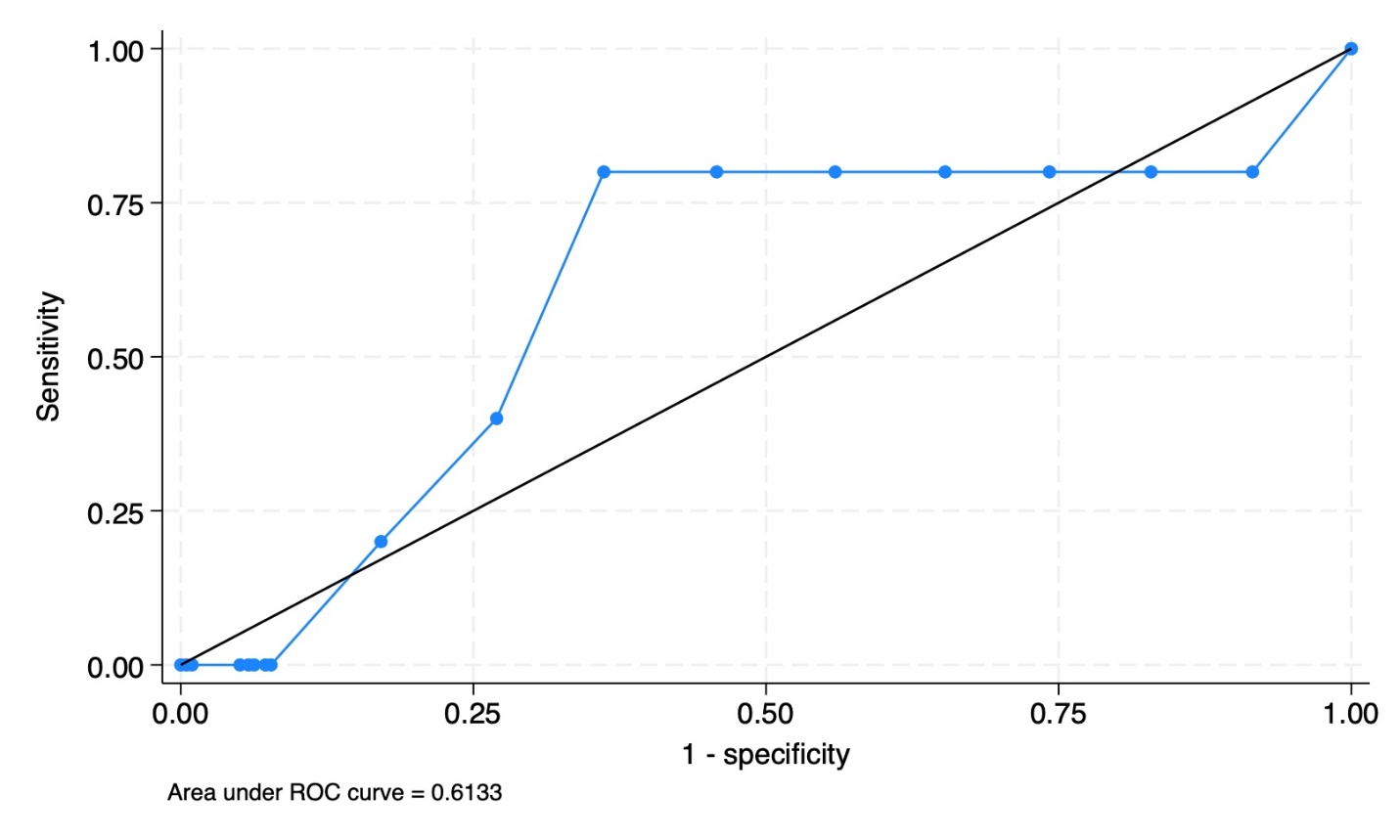


**Supplementary Figure 13**. *Cryptosporidium canis* initial Receiving Operating Characteristic curve.


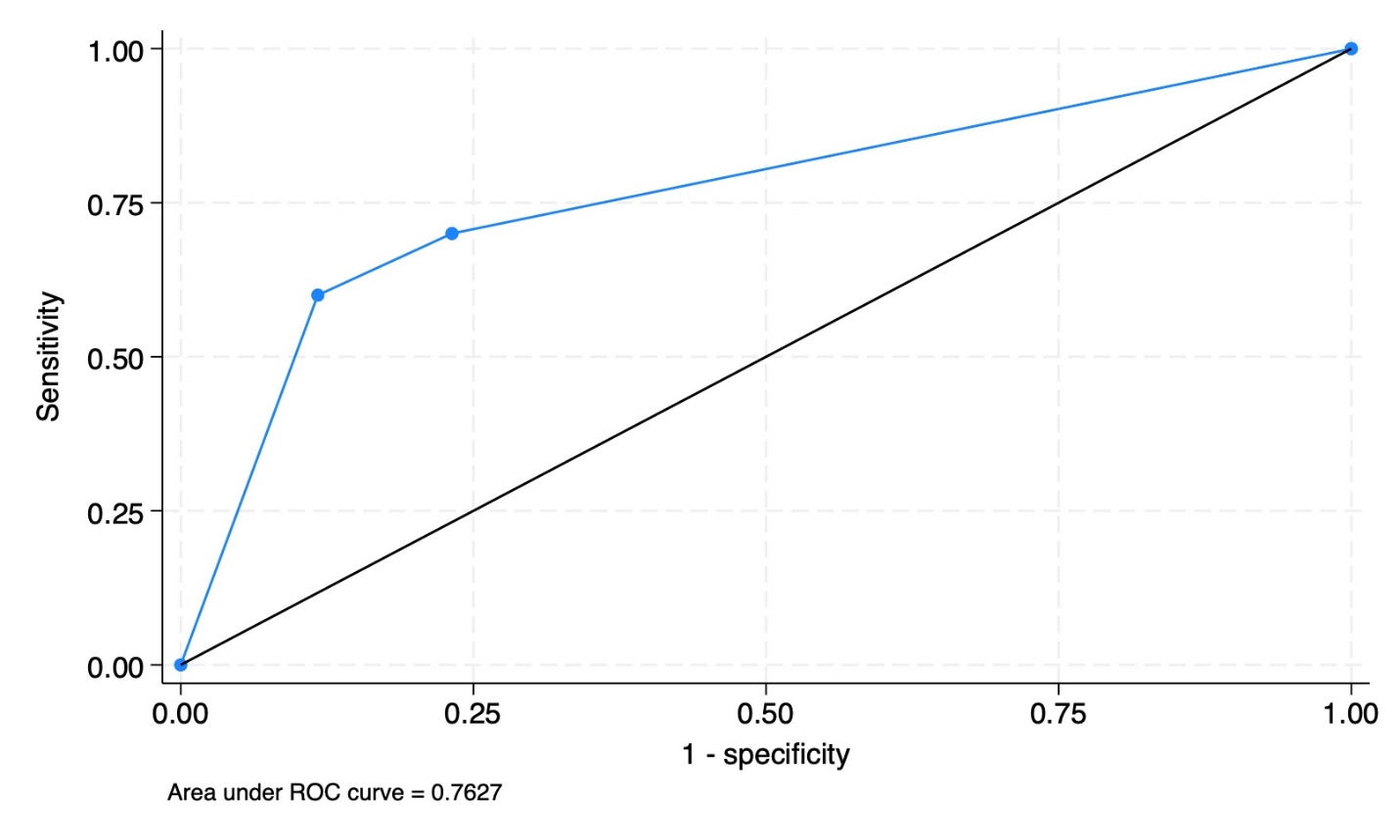


**Supplementary Figure 14**. *Cryptosporidium canis* ten-fold cross validation curve.


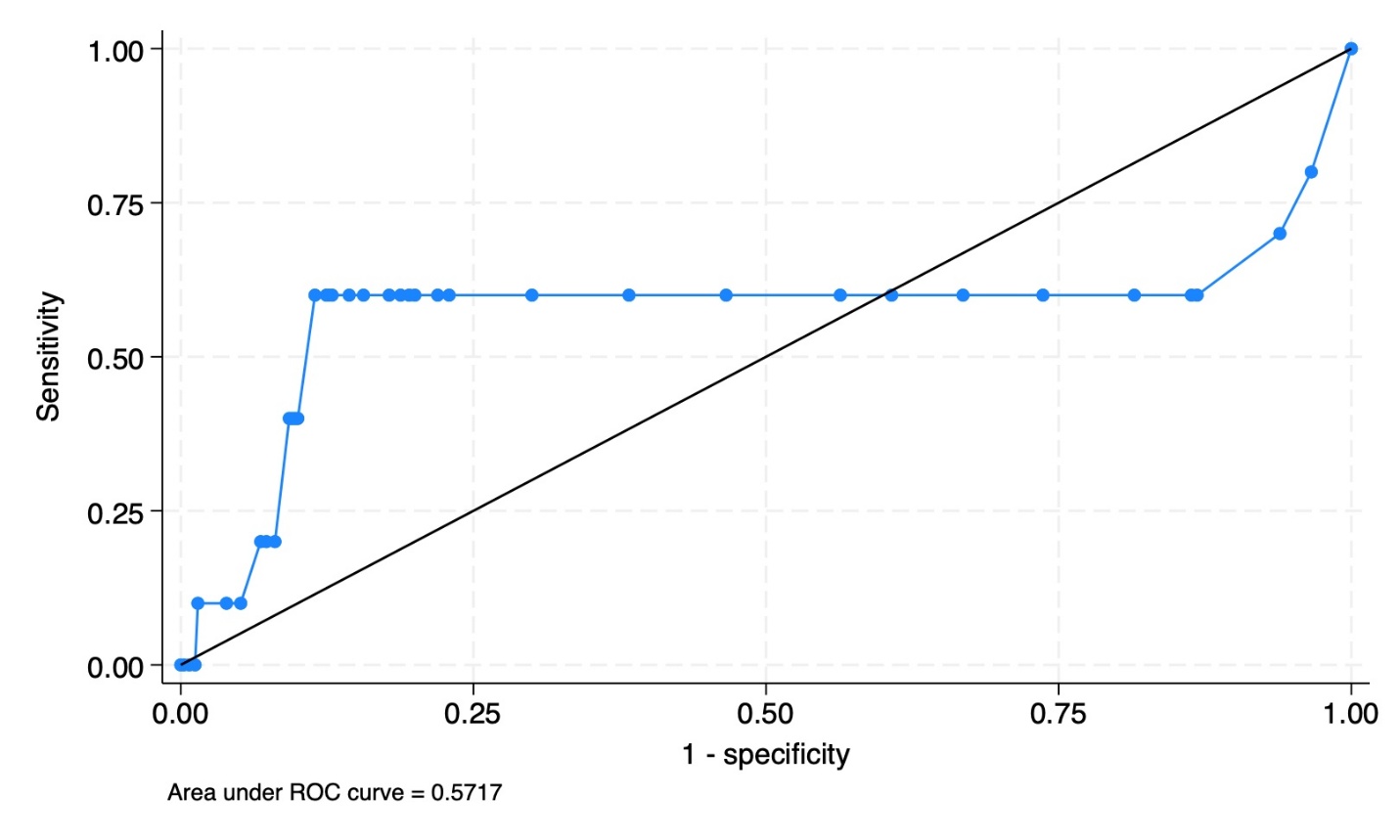


**Supplementary Figure 15**. *Ancylostoma caninum* initial Receiving Operating Characteristic curve.


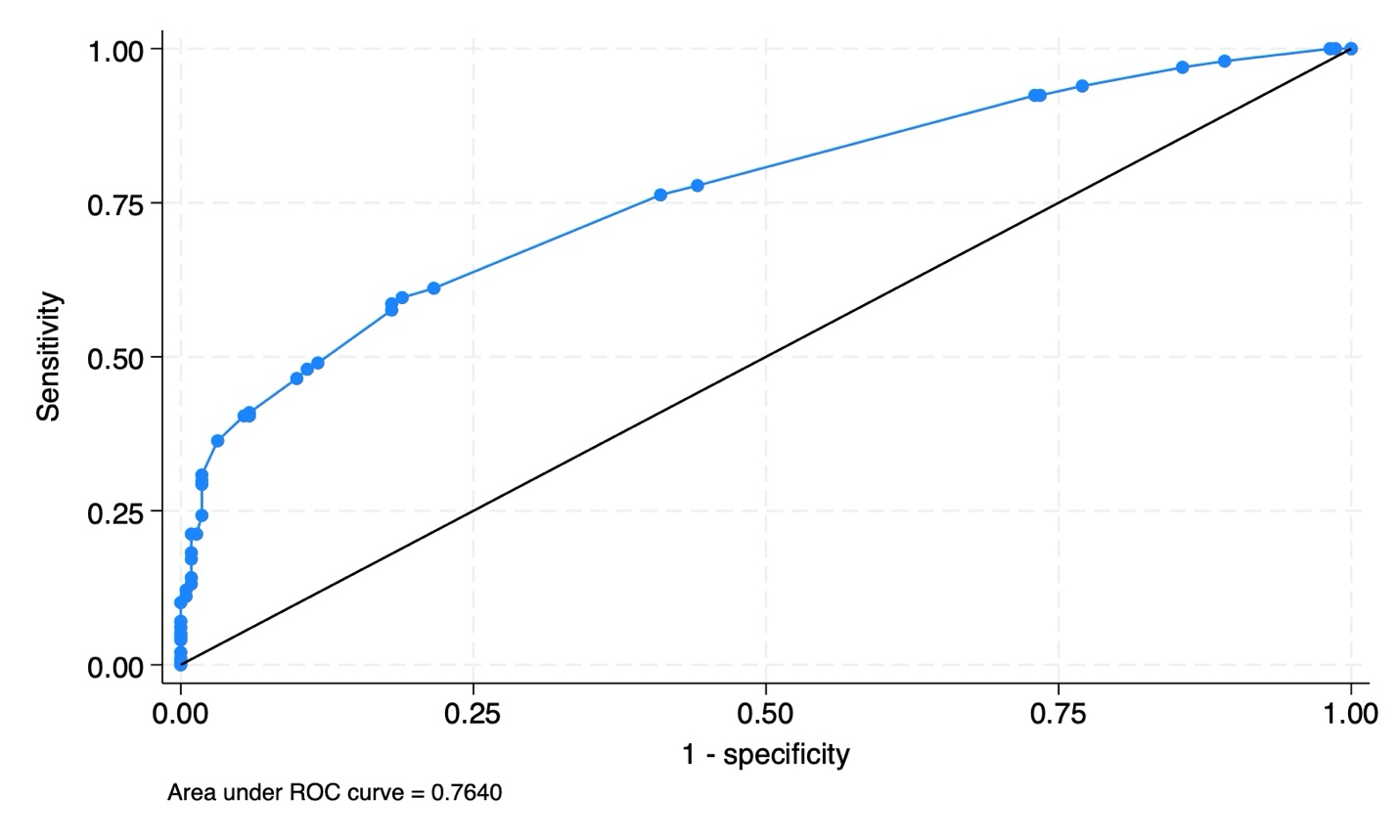


**Supplementary Figure 16**. *Ancylostoma caninum* ten-fold cross validation curve.


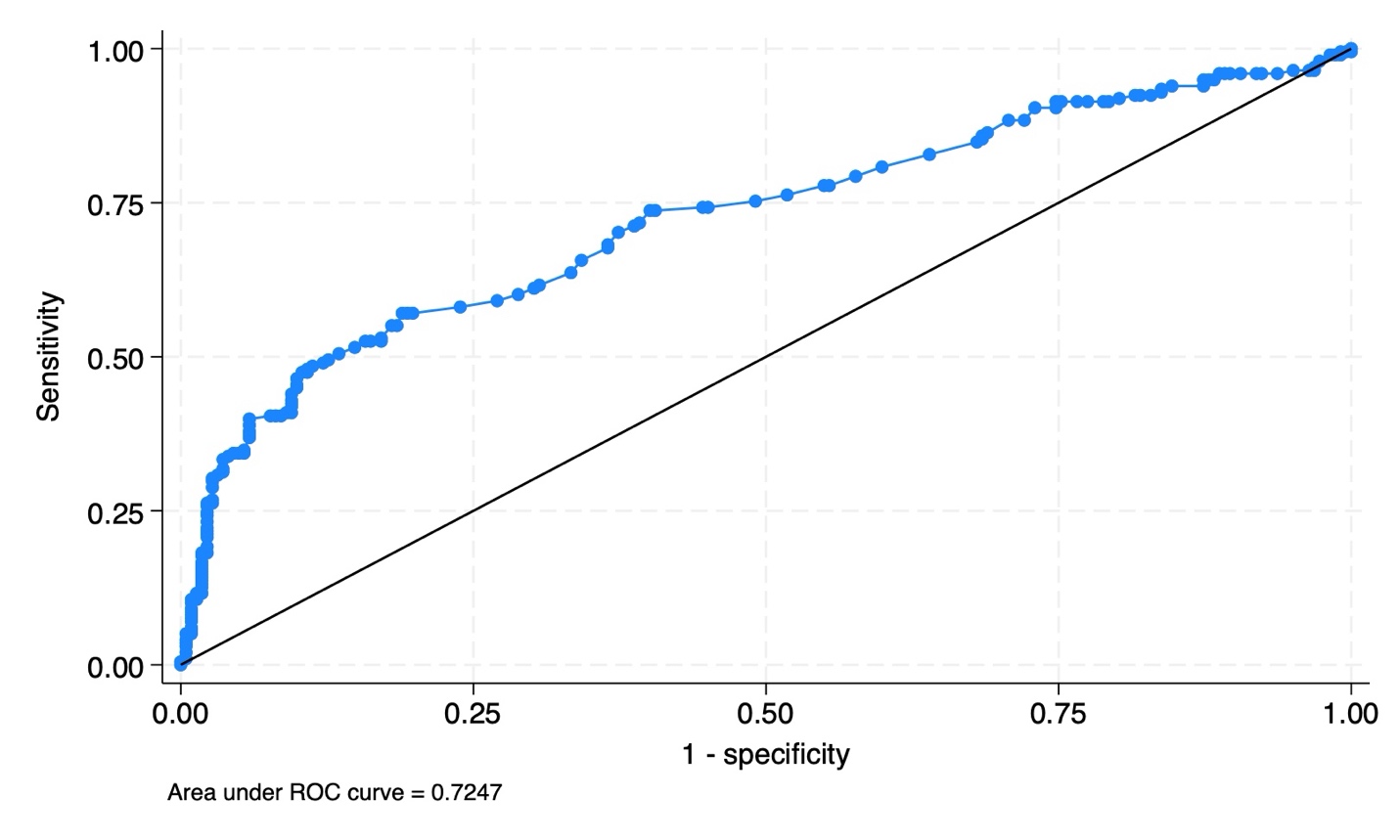


**Supplementary Figure 17**. *Ancylostoma ceylanicum* initial Receiving Operating Characteristic curve.


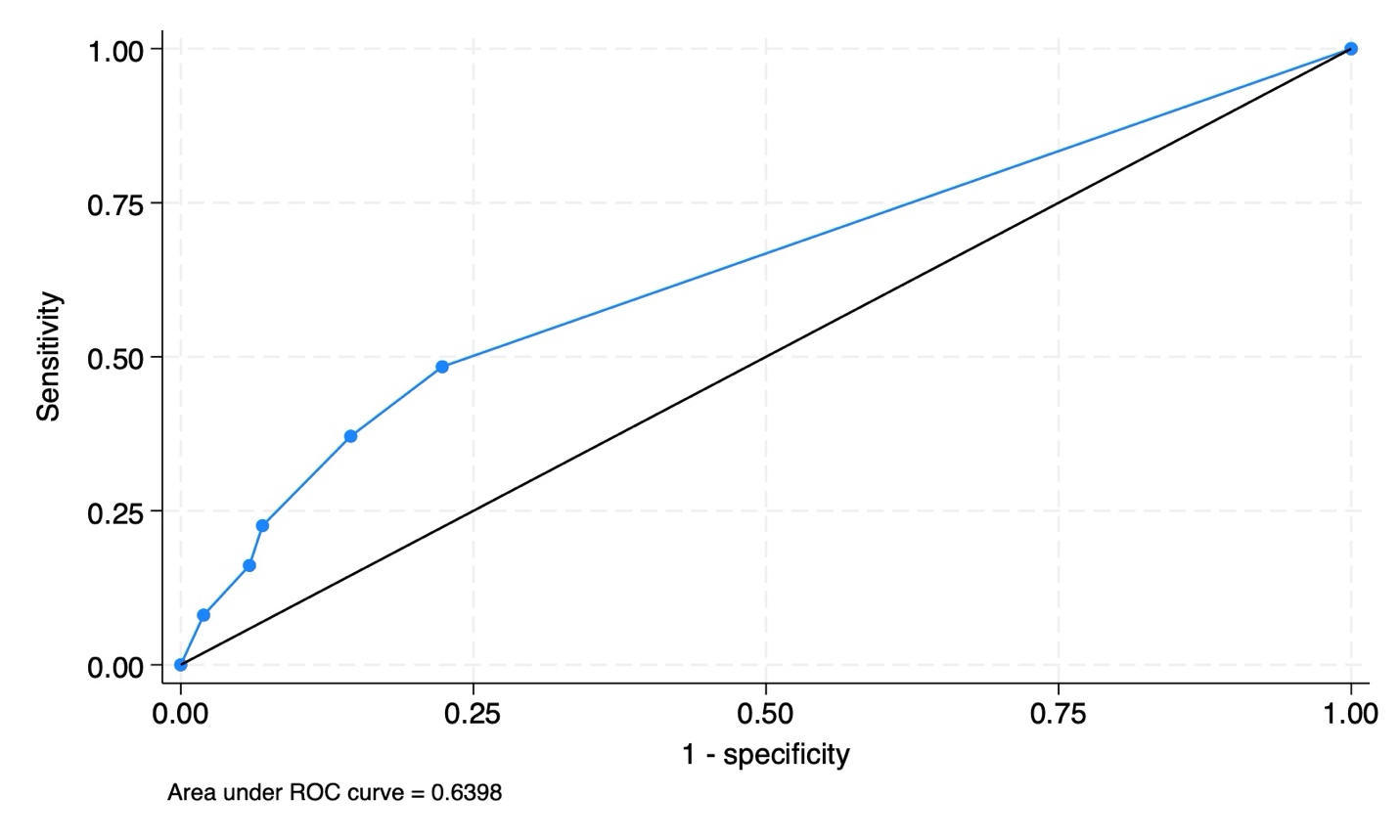


**Supplementary Figure 18**. *Ancylostoma ceylanicum* ten-fold cross validation curve.


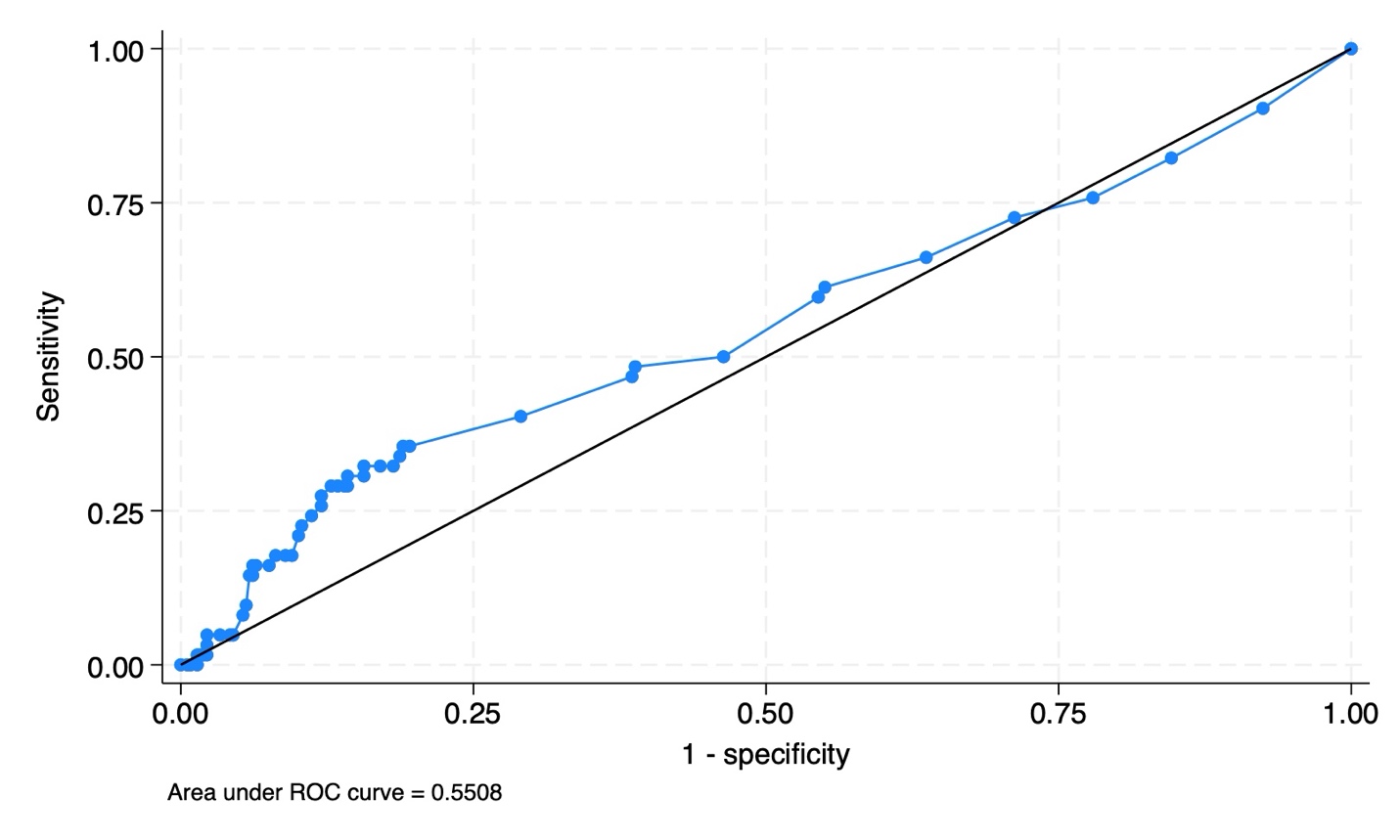

Supplement: Supplementary file 1 — Additional file1 (DOCX 2789 KB) [file 13071_2026_7258_MOESM1_ESM.docx]
